# *Plasmodium* sporozoite phospholipid scramblase interacts with mammalian carbamoyl-phosphate synthetase 1 to infect hepatocytes

**DOI:** 10.1038/s41467-021-27109-7

**Published:** 2021-11-19

**Authors:** Sung-Jae Cha, Min-Sik Kim, Chan Hyun Na, Marcelo Jacobs-Lorena

**Affiliations:** 1 Johns Hopkins Bloomberg School of Public Health, Department of Molecular Microbiology and Immunology and Malaria Research Institute, 615N. Wolfe St., Baltimore, MD 21205 USA; 2 Department of New Biology, Daegu Gyeongbuk Institute of Science and Technology (DGIST), Daegu, 42988 Republic of Korea; 3 Department of Neurology, Institute for Cell Engineering, School of Medicine, Johns Hopkins University, Baltimore, MD 21205 USA

**Keywords:** Parasite biology, Pathogens

## Abstract

After inoculation by the bite of an infected mosquito, *Plasmodium* sporozoites enter the blood stream and infect the liver, where each infected cell produces thousands of merozoites. These in turn, infect red blood cells and cause malaria symptoms. To initiate a productive infection, sporozoites must exit the circulation by traversing the blood lining of the liver vessels after which they infect hepatocytes with unique specificity. We screened a phage display library for peptides that structurally mimic (mimotope) a sporozoite ligand for hepatocyte recognition. We identified HP1 (hepatocyte-binding peptide 1) that mimics a ~50 kDa sporozoite ligand (identified as phospholipid scramblase). Further, we show that HP1 interacts with a ~160 kDa hepatocyte membrane putative receptor (identified as carbamoyl-phosphate synthetase 1). Importantly, immunization of mice with the HP1 peptide partially protects them from infection by the rodent parasite *P*. *berghei*. Moreover, an antibody to the HP1 mimotope inhibits human parasite *P. falciparum* infection of human hepatocytes in culture. The sporozoite ligand for hepatocyte invasion is a potential novel pre-erythrocytic vaccine candidate.

## Abstract (web summary)

After transmission of Plasmodium sporozoites from infected mosquitoes, parasites first infect hepatocytes. Here, Cha et al. identify a sporozoite ligand (phospholipid scramblase) and the hepatocytic receptor (carbamoyl-phosphate synthetase 1) as relevant for hepatocyte invasion and show that an antibody to hepatocyte-binding peptide 1 (HP1), which structurally mimics the sporozoite ligand, partially protects mice from infection.

## Introduction

Malaria infection is initiated by the release from an infected *Anopheles* mosquito of 50–100 *Plasmodium* sporozoites into the skin of a vertebrate host^1^. Thereafter sporozoites migrate through dermal tissues in search of a blood vessel, which they traverse to enter the circulation. Circulating sporozoites must exit in the liver, where they infect hepatocytes, each of which produces thousands of merozoites that are released into the blood circulation and cause disease symptoms^2^. This complex cycle requires specific parasite–host recognition at each stage. Binding of the sporozoite surface circumsporozoite protein (CSP) to liver-specific and highly sulfated glycosaminoglycans (GAGs) protruding into the sinusoidal vessels is the first step for liver recognition and invasion^3,4^. Next, sporozoites glide along the vessel wall and preferentially traverse Kupffer cells via recognition of its CD68 surface receptor^5–8^. After crossing the sinusoid lining, sporozoites specifically invade hepatocytes, no other cell types such as Stellate cells or adipocytes. Thus, we hypothesize that infection requires recognition via interaction of a sporozoite ligand with a hepatocyte receptor. No sporozoite ligand for hepatocyte recognition has been reported. On the other hand, CD81, the scavenger receptor BI (SR-BI), and EphA2 have been proposed as possible hepatocyte receptors for sporozoite invasion^9–11^. The 6-cysteine domain *Plasmodium* protein P36 was proposed to be involved in the SR-BI-dependent pathway^12^. Subsequently, it was determined that CD81 and SR-BI are not sporozoite receptors but rather, are involved in parasitophorous vacuole membrane formation and organization^10^. Furthermore, a recent report shows that EphA2 is not an obligatory receptor for sporozoite hepatocyte interaction^13^. Hepatocyte Aquaporin-9 was recently identified as an important host cell membrane protein for sporozoite permissiveness, however was not characterized as a receptor^14^. Hence, the molecular basis for specific sporozoite–hepatocyte interaction remains unknown. The most advanced available RTS/S malaria pre-erythrocytic vaccine that uses CSP as an antigen only shows moderate protection (~40%)^15^. Identification of a sporozoite ligand for hepatocyte invasion may identify new pre-erythrocytic malaria vaccine targets.

Here we show that a peptide—HP1—identified by the use of a phage display library, specifically binds to hepatocytes and by doing so, inhibits *Plasmodium* sporozoite infection. Further, HP1 is a structural mimic of the sporozoite ligand phospholipid scramblase (PLS) that for infection, interacts with the hepatocyte receptor carbamoyl-phosphate synthetase 1 (CPS1).

## Results

### The hepatocyte-binding peptide HP1 inhibits sporozoite–hepatocyte interaction

**Fig. 1:**
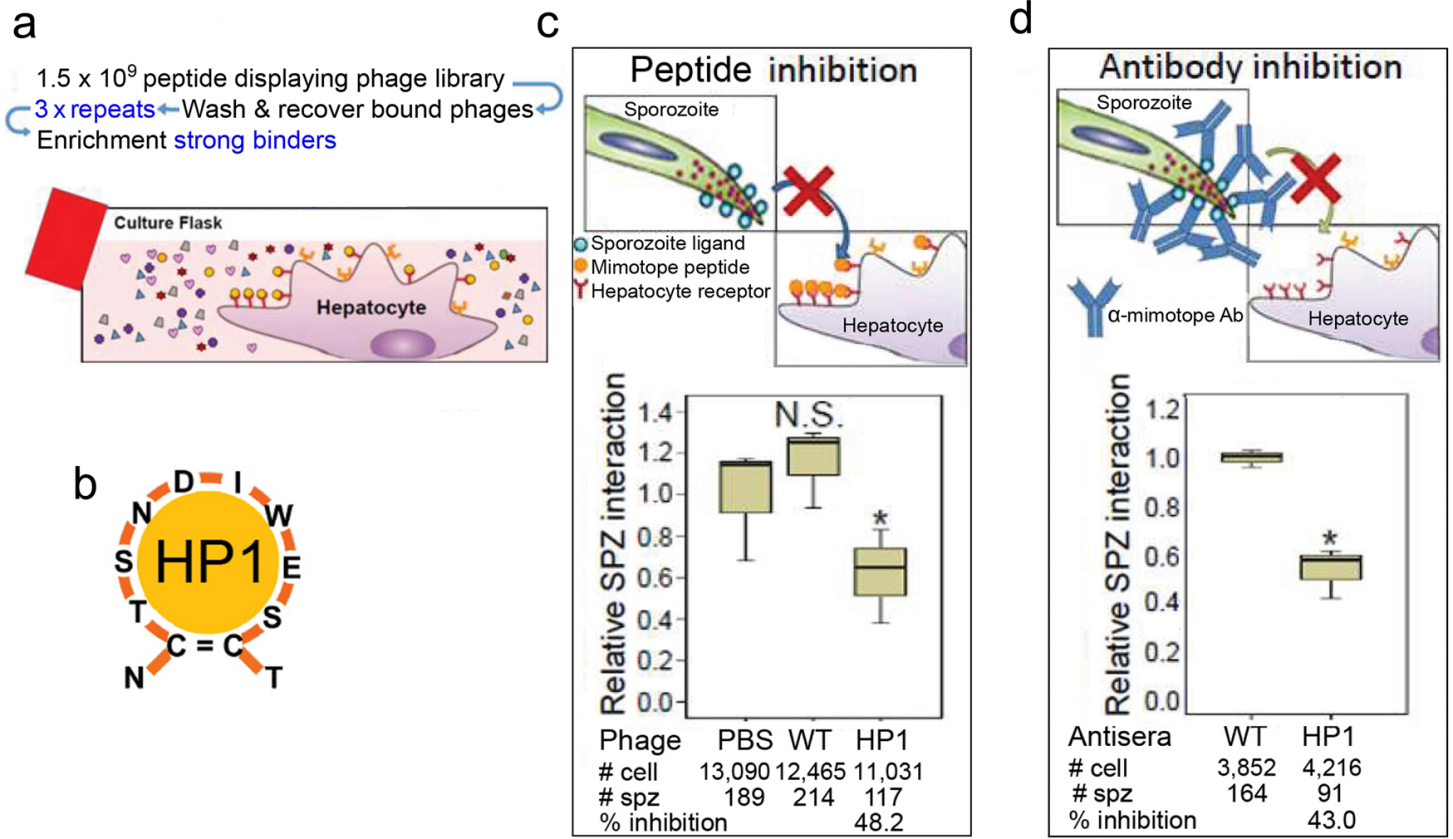
The HP1 peptide mimics a sporozoite ligand. **a** Schematic diagram of the screen for peptides that have a strong affinity to primary mouse hepatocyte surface molecules. The phage display library has a complexity of 1.5 × 10^9^ different peptides. **b** Amino acid sequence and the basic structure of the strongest hepatocyte binder, HP1 peptide. All library peptides have cysteine at positions 2 and 11 that form a disulfide bond and give the peptide conformation. **c** Peptide that mimics the ligand conformation (mimotope) should bind to the hepatocyte receptor and by doing so, inhibit sporozoite–hepatocyte interactions (diagram in the upper panel). Primary mouse hepatocytes were incubated with an HP1 phage or a wild-type phage (non-recombinant; control) and then incubated with *P. berghei* sporozoites. PBS buffer-treated group (no phage) served as another control. Relative sporozoite–hepatocyte interaction was determined by counting the number of sporozoite-bound and -invaded cells/total hepatocyte number (lower panel). Data pooled from three biological replicates. *P = 0.05. NS is not significant. **d** An antibody that recognizes the mimotope should bind to the sporozoite ligand and by doing so, inhibit sporozoite–hepatocyte interactions (diagram in upper panel). *P. berghei* sporozoites were pre-incubated for 10 min with sera from mice that were immunized with HP1 phages and then incubated with primary mouse hepatocytes. Relative sporozoite interaction was determined as described above using WT phage antiserum treatment as a control (lower panel). All box plots show medians, quartiles, minimum, and maximums within the bounds of 1.5 times the interquartile range. % inhibition was calculated using medians. Relative sporozoite interaction was determined by normalizing sporozoite counts compared to the median of the control group, PBS treated, and WT phage antisera treated. Data pooled from three biological replicates. # cell: total hepatocyte number counted; # spz: total sporozoite number counted. *P = 0.05. **c** and **d** Two tail *P*-value by Mann–Whitney *U* test. Source data are provided as a Source Data file.

We used a two-step strategy to investigate the molecular basis for sporozoite–hepatocyte interaction: (1) use a phage peptide display library to select peptides that strongly bind to the hepatocyte surface and (2) determine whether such peptide competitively inhibits sporozoite–hepatocyte interaction. Should competitive inhibition by a small peptide be observed, this would constitute preliminary evidence that the selected peptide binds to a hepatocyte surface molecule that serves as a sporozoite receptor. We screened a phage library displaying 1.5 × 10^9^ different peptides^16^ for peptides with high affinity to primary mouse hepatocytes (Fig. 1a). Of 39 successfully sequenced phages, 17 (43.6%) displayed conserved amino acid sequences. Out of these, five displayed an identical peptide that was named HP1 (Fig. 1b, Supplementary Fig. 1). The recombinant HP1 phage, and the wild-type phage that has no peptide insert as control, were tested for competitive inhibition of sporozoite–hepatocyte interaction. The HP1 phage inhibited sporozoite–hepatocyte interaction by 48% relative to the wild-type phage (Fig. 1c). Additional inhibition assays with sera from mice immunized with the HP1 recombinant phage, or the WT phage as control, revealed that the HP1 antiserum inhibits sporozoite–hepatocyte interactions by 43% (Fig. 1d).

**Fig. 2:**
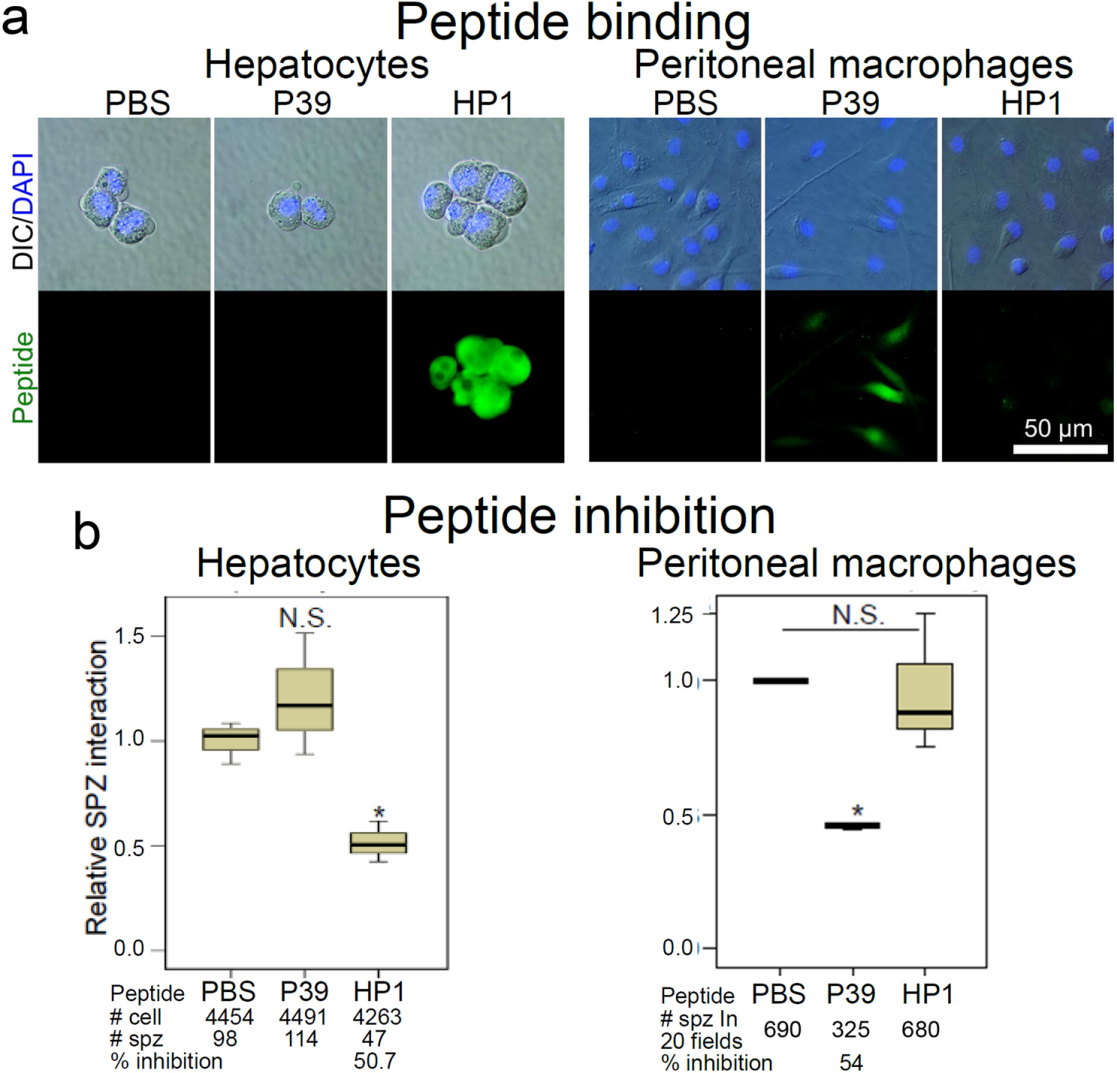
The synthetic HP1 peptide specifically targets *Plasmodium* sporozoite–hepatocyte interaction. **a** A biotin-tagged HP1 peptide was tested for binding to the surface of primary mouse hepatocytes or primary mouse peritoneal macrophages. Peptide binding was detected with Alexa488-conjugated streptavidin. The P39 peptide, which binds to the macrophage surface marker CD68^8^, was used as a negative control for hepatocytes and positive control for macrophages. DIC differential interference contrast microscopy, DAPI: nuclear stain (blue). **b** The HP1 peptide inhibits sporozoite–hepatocyte interaction by 51% relative to the PBS-treated control, while it does not inhibit sporozoite–macrophage interaction (*P = 0.05; NS not significant). The P39 peptide inhibits sporozoite–macrophage interaction but does not inhibit sporozoite–hepatocyte interaction (*P = 0.04; NS not significant). Data pooled from three biological repeats. # cell: total hepatocyte number counted; # spz: total sporozoite number counted. All box plots show quartiles, medians, minimum, and maximums within the bounds of 1.5 times the interquartile range. The % inhibition was calculated using medians. Relative sporozoite interaction was determined by normalizing sporozoite counts comparing it to the mean of the PBS-treated control group. Two tail *P*-value by Mann–Whitney *U* test. *P < 0.05. NS is not significant. Source data are provided as a Source Data file.

**Fig. 3:**
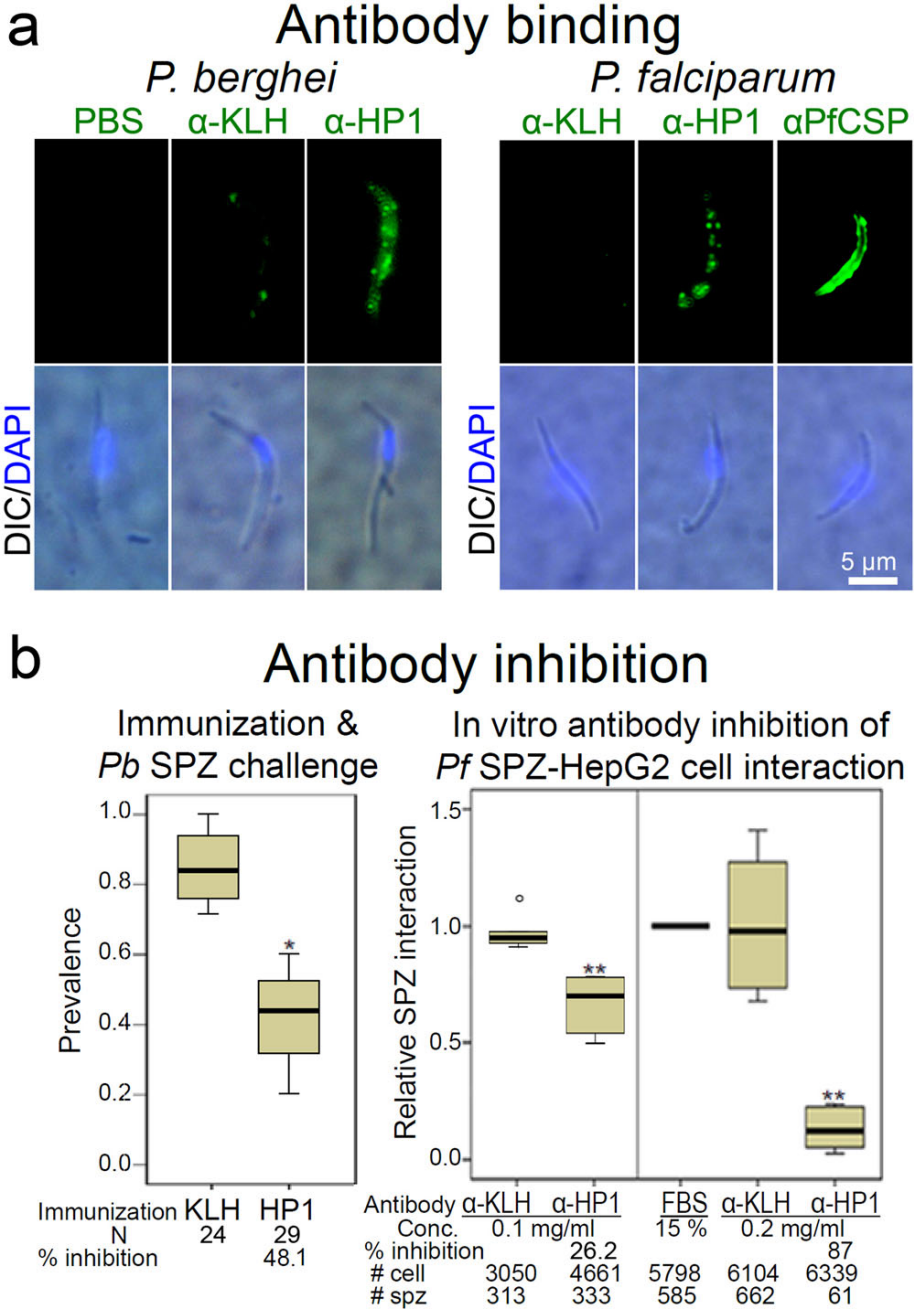
An anti-HP1 peptide antibody binds to the *Plasmodium* sporozoite surface and inhibits sporozoite–hepatocyte interaction. **a** Binding of an anti-HP1 peptide antibody to non-permeabilized *P. berghei* and *P. falciparum* sporozoites was detected with Alexa488-conjugated secondary antibody. **b** **left panel**, Mice were immunized with KLH-conjugated HP1 peptide, and 10 days after the third boost, the mice were challenged by the bite of two infected mosquitoes. Immunization with KLH alone served as a control. The prevalence of mouse infection was determined by Giemsa staining of thin blood smears (*P = 0.02). Data pooled from four biological repeats. *N*: number of mice challenged. **b** **right panel**, *P. falciparum* sporozoites were pre-incubated for 10 min with sera from mice that were immunized with KLH-conjugated HP1 or with non-conjugated KLH, and then incubated with HepG2 human hepatoma cells for 1 h. **P = 0.002 in the left and *P* = 0.004 in the right. Data pooled from two biological repeats. All box plots show quartiles, medians, minimum, and maximums within the bounds of 1.5 times the interquartile range. A dot represents an outlier beyond the upper bound. The % inhibition was calculated using medians. Relative sporozoite interaction was determined by normalizing sporozoite counts compared to the mean of the anti-KLH antibody-treated control group. Two tail *P*-value by Mann–Whitney *U* test. Source data are provided as a Source Data file.

Further experiments were performed with synthetic peptides, instead of recombinant phages, to control for the possibility of steric hindrance by the ~1 μm-long phage particles. The HP1 peptide selectively bound to hepatocytes, but not to macrophages, (Fig. 2a) and inhibited sporozoite–hepatocyte interactions by ~51% relative to the PBS-treated group (Fig. 2b), confirming results of Fig. 1c. The HP1 peptide did not inhibit sporozoite–macrophage interaction. In turn, the unrelated control P39 peptide selectively bound to macrophages, as expected^8^, but not to hepatocytes, and inhibited sporozoite–macrophage interaction, also as expected. Next, mice were immunized with KLH-conjugated HP1 peptide or with non-conjugated KLH as a control. Anti-HP1 sera detected sporozoite surface molecules on unpermeabilized *P. berghei* and *P. falciparum* sporozoites (Fig. 3a). Immunization with the HP1 peptide rendered mice protected from infection with 48% efficacy when challenged with the bite of two infected mosquitos (Fig. 3b, left panel). Immunization with HP1 peptide delayed the pre-patent period compared to the control group, which implies sporozoite liver burden was reduced by anti-HP1 antibodies (Supplementary Fig. 2). Additionally, when human parasite *P. falciparum* sporozoites were incubated with anti-HP1 sera, interaction with HepG2 human hepatoma cells was inhibited in a dose-dependent manner by up to 87% (Fig. 3b, right panel).

### Identification of a sporozoite ligand for hepatocyte interaction

**Fig. 4:**
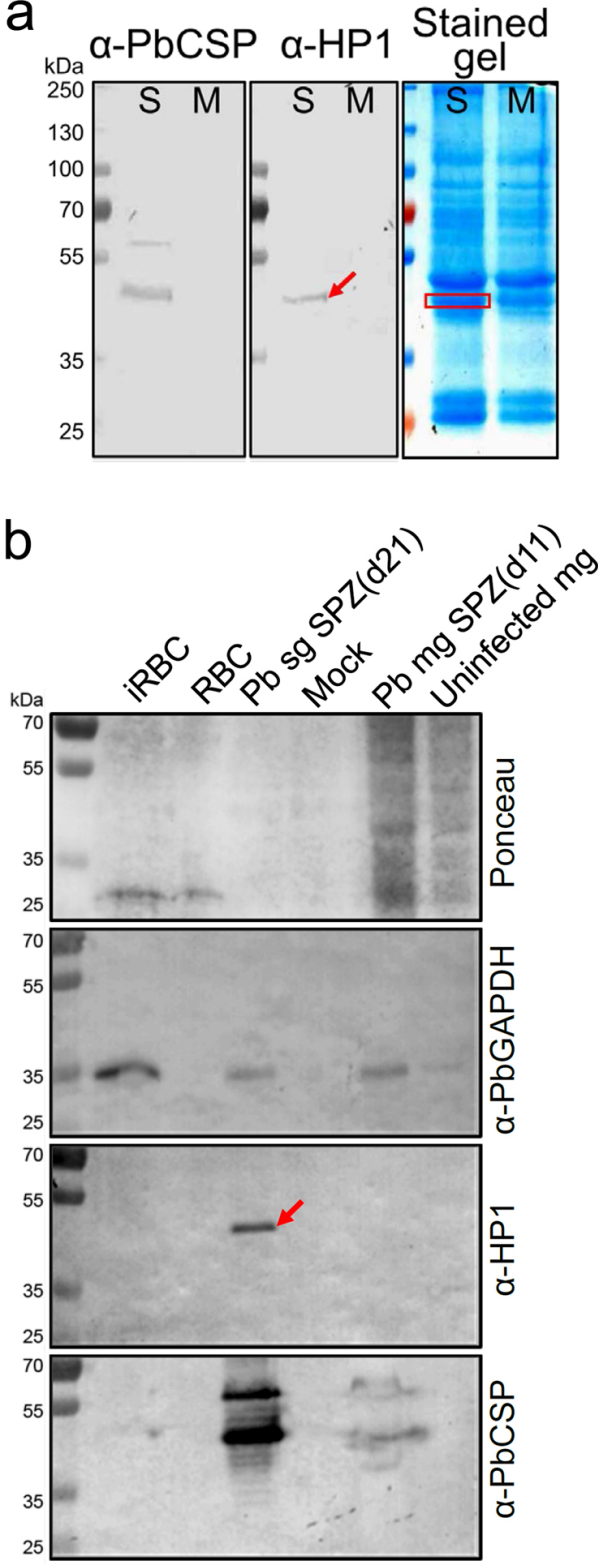
An anti-HP1 antibody recognizes a ~50 kDa sporozoite ligand protein. **a** An anti-HP1 antibody recognizes a ~50 kDa protein band (red arrow) from sporozoites isolated from infected mosquito salivary glands (S) but not from mock preparations from uninfected mosquito salivary glands (M). A red box on the stained gel denotes the gel piece that was cut for mass spectroscopy analysis. **b** Anti-HP1 antibody recognizes a protein specific to mature sporozoites. Lysates of asexual blood-stage parasites (iRBC), purified salivary gland sporozoites (Pb sg SPZ) 21 days after infection, and immature midgut sporozoites (Pb mg SPZ) 11 days after infection were fractionated by SDS–PAGE. Uninfected RBCs, sporozoite preparations from uninfected mosquito salivary glands (Mock), and uninfected midguts were used as controls. Ponceau stain served as a loading reference and an anti-PbGAPDH antibody served as a positive control for *P. berghei* parasite lysates. The anti-HP1 antibody detected a ~50 kDa protein only from salivary gland sporozoites (red arrow). **a** and **b** Each image represents two independent repeats. Source data are provided as a Source Data file.

**Fig. 5:**
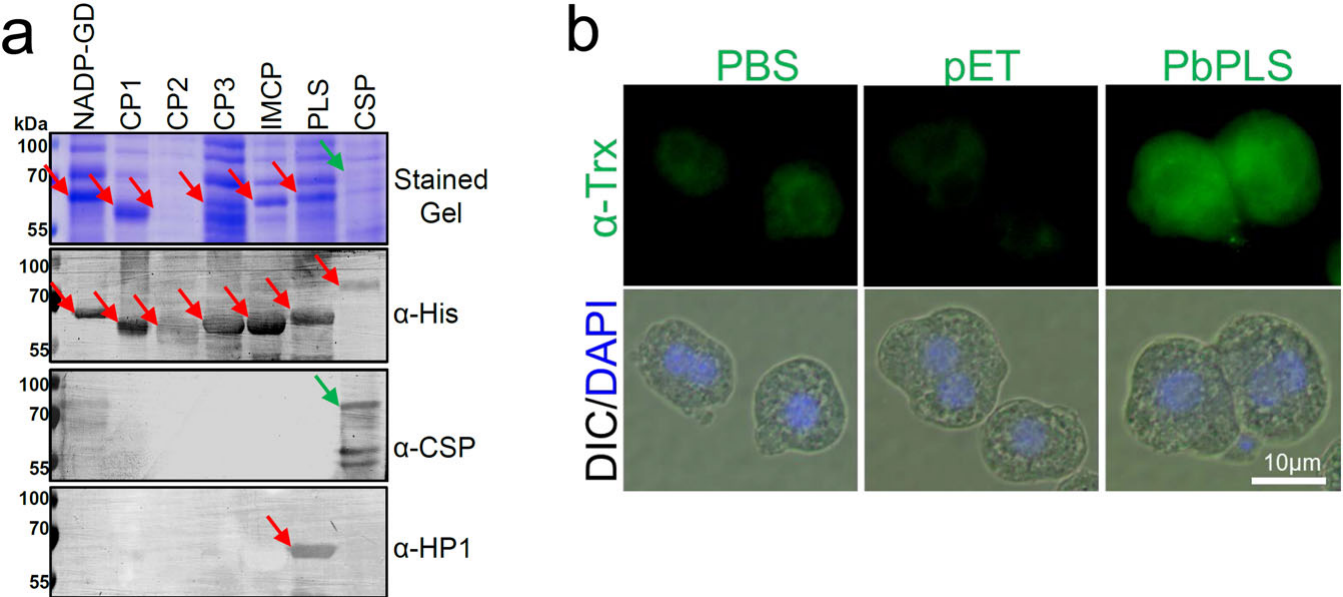
The anti-HP1 antibody recognizes sporozoite scramblase. **a** The seven genes listed in Table 1 were expressed as recombinant proteins and fractionated by SDS–PAGE. Western blotting with an anti-His tag antibody confirmed expression of the recombinant proteins (red arrows) and an anti-CSP antibody confirmed CSP expression (green arrow). Importantly, the anti-HP1 antibody only recognized the scramblase (PLS) protein (red arrow). **b** the PLS protein binds to hepatocytes. Recombinant PLS, pET tag protein, or PBS were incubated with primary mouse hepatocytes. Protein binding was visualized with an anti-thioredoxin-tag antibody. **a** and **b** Each image represents two independent repeats. DIC differential interference contrast microscopy; DAPI nuclear stain (blue). Source data are provided as a Source Data file.

**Fig. 6:**
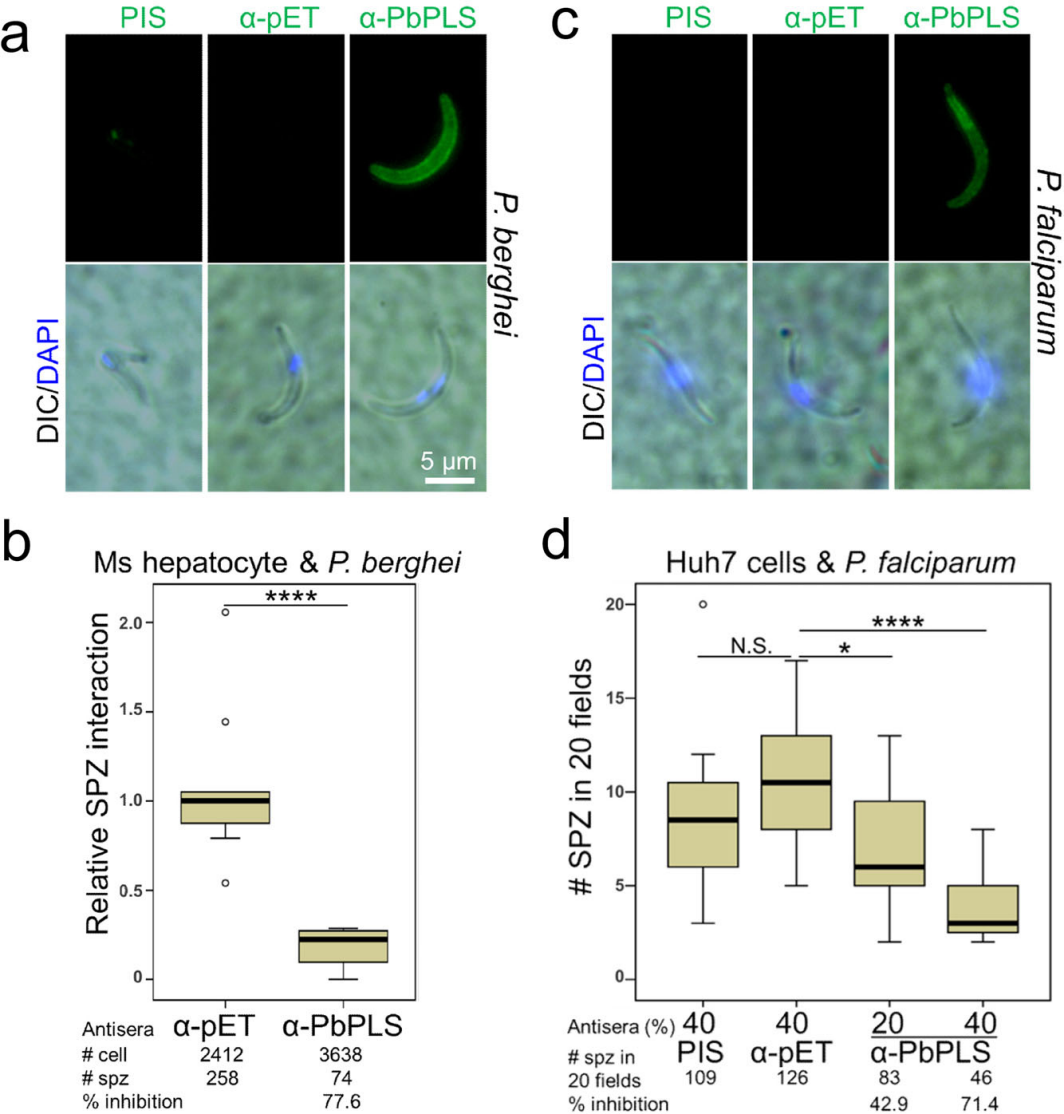
The anti-PbPLS antibody binds to the sporozoite surface and inhibits sporozoite–hepatocyte interaction. **a** Immunofluorescent assays show that an anti-PbPLS antibody binds to unpermeabilized *P. berghei* sporozoites. Antibody binding was visualized with Alexa488-conjugated secondary antibody. Preimmune sera (PIS) or anti-pET tag antibody do not bind. **b** An anti-PbPLS serum strongly inhibits *P. berghei* sporozoite–primary mouse (Ms) hepatocyte interaction (****P < 0.0001). # cell: total hepatocyte number counted; # spz: total sporozoite number counted. **c** An anti-PbPLS antibody binds to unpermeabilized *P. falciparum* sporozoites. **d** An anti-PbPLS antibody inhibits *P. falciparum* sporozoite-human hepatoma Huh7 cell interaction in a dose-dependent manner (*P = 0.02; ****P < 0.0001; NS not significant). All box plots show quartiles, medians, and maximum within the bounds of 1.5 times interquartile range. Dots represent outliers beyond the upper and lower bounds. The % inhibition was calculated using medians. Relative sporozoite interaction was determined by normalizing sporozoite counts comparing to the mean of the anti-pET antibody-treated control group. Data pooled from two independent assays. Two tail *P*-value by Mann–Whitney *U* test. **a** and **c** Each image represents two independent repeats. Source data are provided as a Source Data file.

The following experiments were performed to test the hypothesis that the HP1 peptide is a mimotope (shares conformation) of a sporozoite surface protein that functions in the interaction with liver cells. This hypothesis predicts that an anti-HP1 antibody also recognizes a sporozoite ligand (see Fig. 1d diagram). Western blotting of *P. berghei* sporozoite lysates with an anti-HP1 antibody detected a ~50 kDa protein, a putative sporozoite ligand (Fig. 4a). Although this ~50 kDa band is at a similar position as a cleaved CSP band, the anti-HP1 antibody did not detect a band at the position of the larger uncleaved CSP protein, indicating that the HP1 peptide is not a CSP mimotope. This was confirmed by determining that the anti-HP1 antibody does not recognize an immunoprecipitated CSP protein (Supplementary Fig. 3). Additional Western blotting assays show that the expression of the ~50 kDa putative sporozoite ligand is limited to mature salivary gland sporozoites (Fig. 4b). Mass spectroscopic analysis of the ~50 kDa sporozoite proteins from a gel piece shown in Fig. 4a (stained gel, red box) identified seven sporozoite-specific proteins, including CSP (Table 1). PlasmoDB (plasmodb.org) predicts that the six proteins and CSP are expressed on the sporozoite surface. These proteins were expressed using a bacterial expression system (Fig. 5a). Expression was confirmed using an anti-His tag antibody (red arrows in the second panel) and anti-CSP antibody (green arrow in the third panel). Importantly, the anti-HP1 antibody recognized only one of the proteins (red arrow in the bottom panel), PLS. Specific recognition of the recombinant PbPLS by an anti-HP1 antibody was confirmed with ELISA assays (Supplementary Fig. 4). The PLS role as a sporozoite ligand for hepatocyte interaction is supported by the observation that recombinant PLS binds to the surface of hepatocytes (Fig. 5b). To test whether an anti-PLS antibody inhibits sporozoite–hepatocyte interaction, mice were immunized with the recombinant PbPLS protein or with the empty pET tag protein as a control. Anti-PbPLS antibodies recognized the HP1 peptide (Supplementary Fig. 5) and Western blots from sporozoite lysates probed with the anti-PbPLS antibody and the anti-HP1 antibody detected proteins with the same electrophoretic mobility (Supplementary Fig. 6). Importantly, anti-PbPLS antibodies bound to the surface of unpermeabilized *P. berghei* sporozoites (Fig. 6a), and by doing so inhibited sporozoite–primary mouse hepatocyte interaction by 78% (Fig. 6b). Additionally, anti-PbPLS antibodies cross-reacted with *P. falciparum* sporozoite surface PLS (Fig. 6c) and similarly inhibited sporozoite–Huh7 human hepatoma cell interaction in a dose-dependent manner (Fig. 6d).

**Table 1:** Mass spectroscopy analysis of a gel piece (red box in Fig. 4a) identified seven sporozoite surface proteins.

PlasmoDB ID	# peptide	Spz surface reported	GPI anchor	Master protein descriptions
PBANKA_0510200	1	Yes	No	Conserved *Plasmodium* protein, unknown function
PBANKA_1422900	2	Yes	No	Conserved *Plasmodium* protein, unknown function
PBANKA_1034500	2	Yes	No	Conserved *Plasmodium* protein, unknown function
PBANKA_1240600	2	Yes	No	Inner membrane complex protein 1 g, putative
PBANKA_1026200	13	Yes	No	NADP-specific glutamate dehydrogenase, putative
PBANKA_0506900	1	Yes	No	Phospholipid scramblase, putative
PBANKA_0403200	1	Yes	Yes	CSP

### Identification of a hepatocyte receptor for sporozoite interaction

**Fig. 7:**
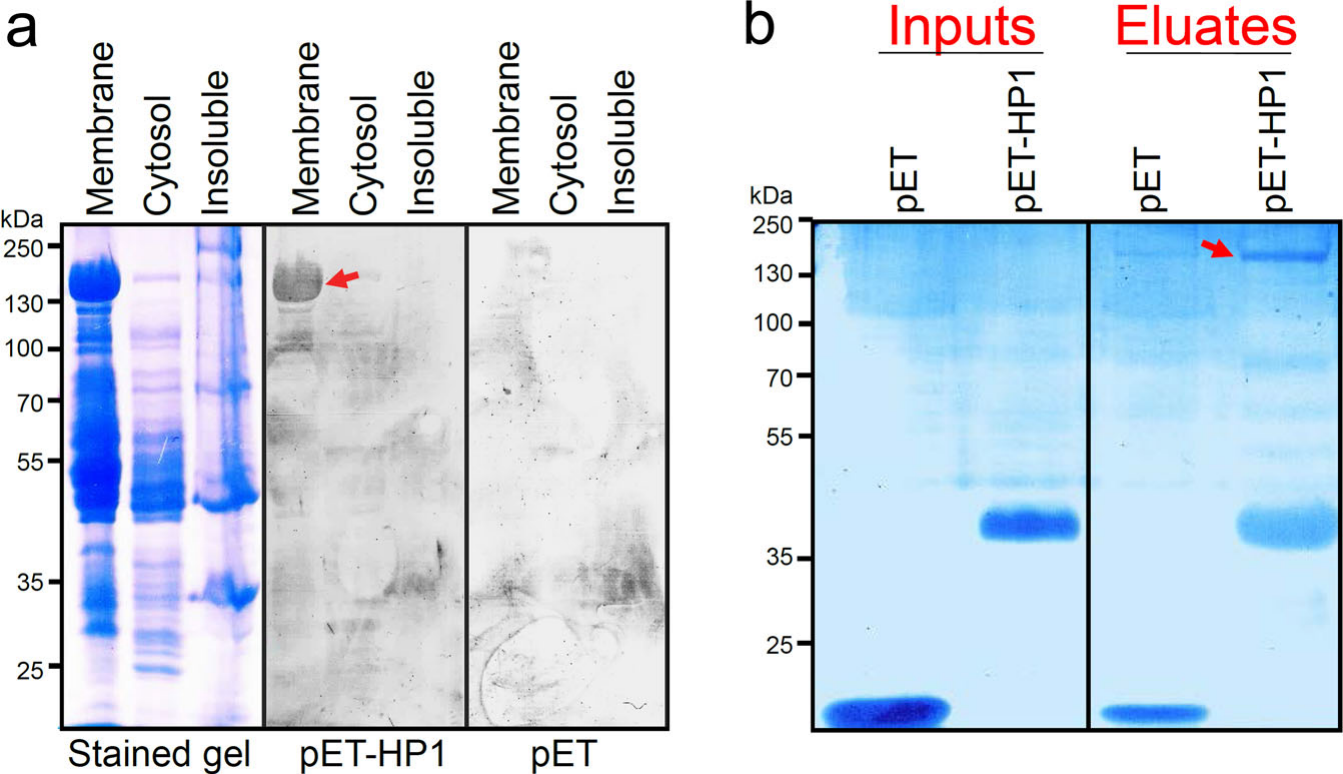
The HP1 peptide and recombinant PLS bind to a ~160 kDa hepatocyte membrane protein. **a** Far-Western blotting assay of mouse primary hepatocyte fractions using recombinant pET-HP1 peptide or 19 kDa empty pET tag protein as a control. Recombinant pET-HP1 binding to a ~160 kDa hepatocyte membrane protein (red arrow) was detected with an anti-tag antibody. **b** Primary mouse hepatocyte membrane proteins were incubated either with empty recombinant pET tag or recombinant pET-HP1 peptide. The pET-HP1 peptide specifically pulled down a ~160 kDa mouse hepatocyte membrane protein (red arrow). Coomassie blue-stained gel. **a** and **b** Each image represents two independent repeats. Source data are provided as a Source Data file.

**Fig. 8:**
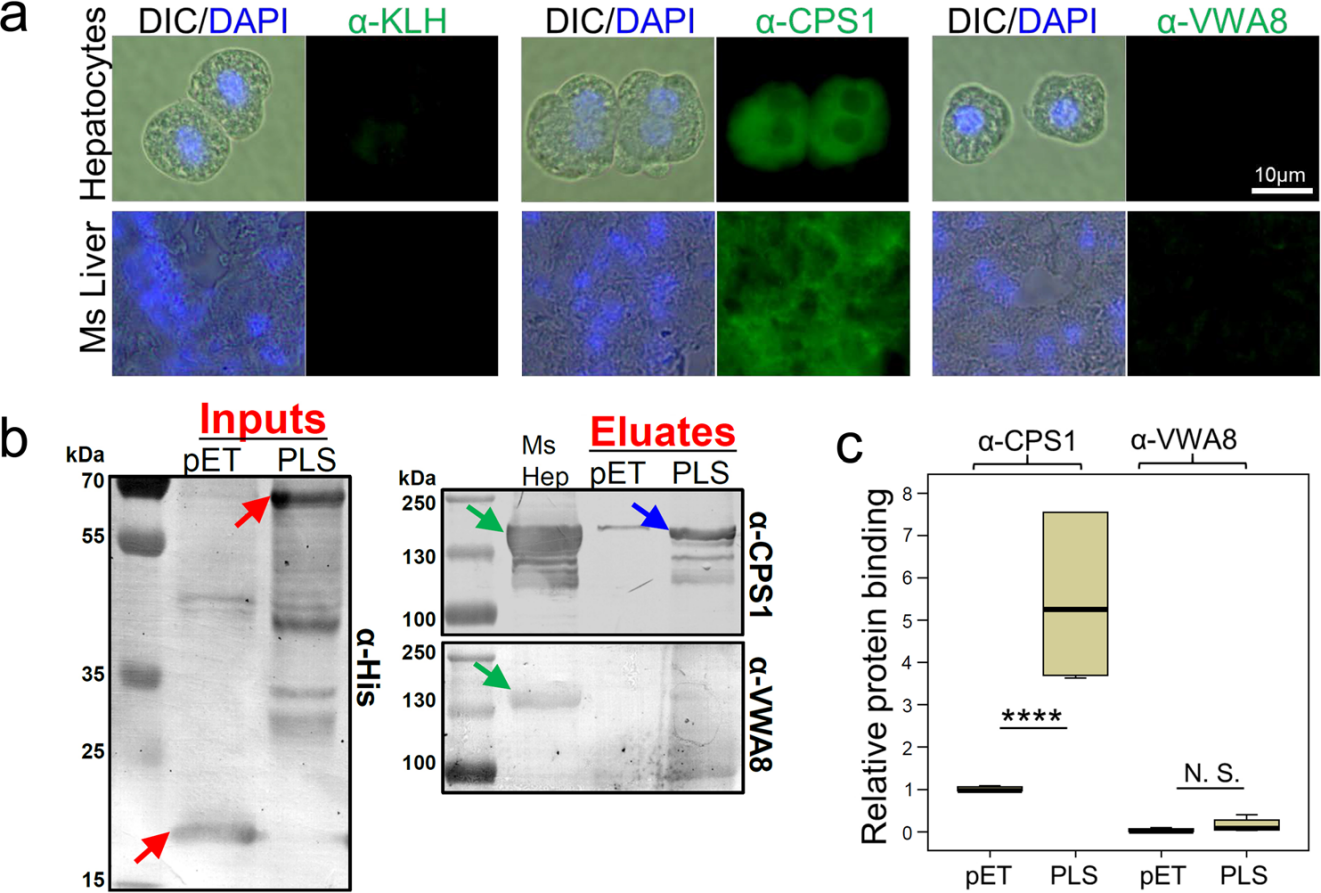
Sporozoite PLS binds to hepatocyte CPS1. **a** Immunofluorescence assays show that CPS1 is present on the surface of non-permeabilized primary mouse hepatocytes. DIC differential interference contrast microscopy, DAPI nuclear stain (blue). Ms liver mouse liver sections. **b** Tagged recombinant sporozoite PLS and pET tag proteins (red arrows) were attached to S-tag agarose beads and incubated with lysates of primary mouse hepatocytes (Ms Hep). Bound hepatocyte proteins were eluted into loading buffer and fractionated by SDS–PAGE. Western blotting (antibody indicated to the right of the panels) shows that liver CPS1 binds to the recombinant sporozoite PLS protein (blue arrow). Hepatocyte lysate was used as a positive control to verify anti-CPS1 and anti-vWA8 antibody binding (green arrows). **c** An ELISA assay shows that the recombinant sporozoite PbPLS interacts with the recombinant mouse hepatocyte CPS1. Ni-coated ELISA plate was incubated overnight with recombinant sporozoite PbPLS or pET tag protein as a control. After blocking, mouse hepatocyte lysate was added to each well. Hepatocyte CPS1 or VWA8 protein binding was determined using anti-CPS1 or anti-VWA8 antibodies, alkaline phosphatase-conjugated secondary antibody, and colorimetric substrate. The protein-binding intensity was normalized to anti-S-tag antibody binding, which determines the quantity of the recombinant protein bound to the plate (****P < 0.0001; NS not significant). Data pooled from two independent experiments. All box plots show quartiles, medians, and maximum within the bounds of 1.5 times interquartile range. Two tail *P-*value was calculated with the one-way ANOVA TukeyHSD test**. a** and **b** Each image represents two independent repeats. Source data are provided as a Source Data file.

To identify the HP1-binding target on the hepatocyte surface, a recombinant tetrameric HP1 peptide was produced using a bacterial expression system (Supplementary Fig. 7a). We could not use a biotin-tagged HP1 peptide because hepatocytes have many endogenously biotinylated proteins. The recombinant pET-HP1 peptide bound to mouse hepatocytes (Supplementary Fig. 7b) and inhibited *P. falciparum* sporozoite–HepG2 human hepatoma cell interaction in a dose-dependent manner (Supplementary Fig. 7c). Hepatocyte membrane, cytosolic, and insoluble fractions were fractionated by SDS–PAGE followed by far-Western blotting with the recombinant pET-HP1 peptide, or with empty pET tag protein as a control. This assay identified a ~160 kDa hepatocyte membrane protein as a target of the recombinant pET-HP1 peptide binding (Fig. 7a). As an alternative approach, pull-down of primary mouse hepatocyte membrane proteins with the recombinant pET-HP1 peptide identified a protein of the same size (Fig. 7b, red arrow). Mass spectroscopy analysis of the ~160 kDa proteins from the gel in Fig. 7b (red arrow) identified five candidate hepatocyte membrane proteins (Table 2). Of these, only CPS1 has been shown to localize to the cell surface^17^. We confirmed that CPS1, an abundant liver protein, is exposed on the surface of primary mouse hepatocytes (Fig. 8a). Although another candidate VWA8 (von Willebrand factor type A domain-containing protein; Table 2) has a well-known extracellular protein domain (vWA)^18^, its presence on the hepatocyte surface could not be detected (Fig. 8a). In a complementary approach, we pulled down hepatocyte proteins with the recombinant sporozoite PLS protein. Western blotting of the eluates determined that PLS pulls down hepatocyte CPS1 (Fig. 8b). Further, direct interaction between the recombinant PbPLS and hepatocyte CPS1 proteins was verified with ELISA assays (Fig. 8c).

**Table 2:** Mass spectroscopy analysis of a gel band (red arrow in Fig. 7b) identified five hepatocyte putative membrane proteins.

Uniprot ID	# peptide	Cell surface reported	Transmembrane domain	Description
Q8C196	279	Yes	Yes	Carbamoyl-phosphate synthase, Cps1
Q8CC88	111	No	Yes	von Willebrand factor A domain-containing protein 8, Vwa8
Q9DBM2	81	No	Yes	Peroxisomal bifunctional enzyme, Ehhadh
Q6PB66	105	No	Yes	Leucine-rich PPR motif-containing protein, Lrpprc
F8WHL2	43	No	Yes	Coatomer subunit alpha, Copa

## Discussion

Previously, our group pioneered the use of phage peptide display library screening to investigate the molecular basis of *Plasmodium* ookinete–mosquito midgut interaction, *Plasmodium* fertilization, sporozoite–mosquito salivary gland interaction, and sporozoite–Kupffer cell interaction^8,17,19–23^. An important feature of this approach is that it does not require a priori knowledge of any property of the interacting partner proteins. In this study, we performed two independent screens of a phage library to select peptides that bind to primary mouse hepatocytes. Both screens selected the same HP1 peptide with an overall 44% frequency, indicating that biopannings reliably lead to enrichment of the strongest binder.

Our attempt to immunoprecipitate the ~50 kDa sporozoite protein recognized by an anti-HP1 antibody was not successful, perhaps because of poor solubility of the PLS membrane lipid raft protein that scrambles membrane phospholipids^24,25^. Recently the scramblase activity of *P. falciparum* PLS was confirmed, and deletion of the gene did not affect blood-stage parasite growth^26^. PLS, whose presence on the surface of the salivary gland–sporozoites had been reported^27^, may not use enzyme activity for interaction with its liver receptor because the enzyme–substrate pocket resides within the parasite plasma membrane, not in the extracellular domain^28^. Other functional roles attributed to PLS are the interaction of this surface protein with extracellular signaling molecules^29^ and mediation of hepatitis C virus entry into the host cell by interacting with viral envelop proteins^30^. As is the case for PLS and CPS1, our previous studies found that enolase on the surface of ookinetes and GAPDH on the surface of sporozoites also perform moonlighting functions as parasite ligands^19,23^.

PLS is well conserved among *Plasmodium* species (~75% identity) and poorly conserved with mammalian proteins (~21% identity between *P. falciparum* and human PLS). Furthermore, PfPLS has low polymorphism: only two non-synonymous SNPs were identified, with 1% frequency each out of 219 sequenced strains (plasmodb.org). This low polymorphism may be due to the short exposure time of this sporozoite-stage specific protein to the host immune system. Moreover, low polymorphism bodes well for the potential use of PLS as an additional vaccine antigen. *Plasmodium* CSP, the most abundant sporozoite surface protein and antigen of present vaccine trials, has 46 non-synonymous SNPs out of 213 sequenced strains, some alleles occurring with 46% frequency (plasmodb.org). Although the latest R21 malaria vaccine on trial, which uses the central repeat and C-terminus of the PfCSP, reported ~75% of protective efficacy^31^, large-scale field trial may encounter reduction of protective efficacy due to the polymorphisms in the C-terminal region, as was the case for the RTS,S malaria vaccine^32^.

CPS1 is a putative hepatocyte receptor because it physically interacts with the sporozoite ligand PLS and because binding of the HP1 mimotope to CPS1 inhibits sporozoite–hepatocyte interaction. CPS1 is reported to be a mitochondrial enzyme involved in the urea cycle. However, it is also constitutively secreted and aggregated on the hepatocyte surface^33^. Functional role of the hepatocyte CPS1 as a sporozoite receptor has never been explored and appears to have this moonlighting function. CPS1 secretion increases upon liver injury and circulating CPS1 binds to macrophages, acting as a protective cytokine that inhibits inflammation^33^. Prior to the final productive infection, *Plasmodium* sporozoites migrate through several hepatocytes by breaching their plasma membrane^34^. This suggests that hepatocyte injury activates CPS1 secretion and inhibits liver macrophage inflammation, in this way supporting early liver stage parasite growth.

In summary, we identified a hepatocyte-binding peptide—HP1, that interacts with the CPS1 hepatocyte surface protein (putative receptor) and whose conformation mimics the sporozoite PLS surface protein (putative ligand). Hepatocyte CPS1 directly interacts with recombinant sporozoite PLS. Importantly, interference with this interaction represses sporozoite infectivity and immunization with HP1 partially protects mice from *Plasmodium* infection. Targeting sporozoite PLS–hepatocyte CPS1 interaction should be explored as a novel malaria control strategy.

## Methods

### Animal ethics protocol

This study was performed in strict accordance with the recommendations from the Guide for Care and Use of Laboratory Animals of the National Institutes of Health (NIH). The animal use was done in accordance with protocol MO21H18 approved by the Johns Hopkins University Animal Care and Use Committee

### Cell culture and sporozoite isolation

For isolation of mouse hepatocytes, a ~20 g male or female C57BL/6J mouse was perfused through the portal vein with 0.05% collagenase type IV for 15 min at 37 °C. After perfusion, liver cells were dissociated, and non-parenchymal cells were removed with 3-min centrifugation at 50 × *g*, repeated three times. The hepatocyte-containing pellet was resuspended in DMEM medium and dead cells were removed by 25% Percoll centrifugation at 500 × *g* for 10 min. Live hepatocytes in 25% Percoll were transferred to fresh DMEM medium supplemented with 10% heat-inactivated fetal bovine serum (hiFBS). Primary mouse peritoneal macrophages were isolated from a C57BL/6J mouse as previously described^22^ and incubated in the same medium. Briefly, 4 ml of PBS was injected into the peritoneum and then carefully retrieved using a 20 G needle. After centrifugation at 500 × *g* for 4 min in a 15 ml tube, the cell pellet was resuspended in 2 ml Ack lysis buffer (Gibco) to remove RBC. After centrifugation at 500 × *g* for 4 min, peritoneal cells were resuspended in DMEM medium with 10% hiFBS and cultured in eight-well chamber slides (Thermo Scientific). All procedures were executed in accordance with a protocol approved by the Johns Hopkins University Animal Care and Use Committee. Mouse rooms are maintained at 30–70% relative humidity and a temperature of 18–26 °C (64–79 °F) providing 14 h light/10 h dark. Human hepatoma cells, HepG2 and Huh7, were maintained in the DMEM medium with 10% hiFBS. *P. falciparum* (NF54) or *P. berghei* (tdTomato-expressing ANKA 2.34)^35^ sporozoites were isolated from infected female *An. stephensi* mosquitoes by dissecting salivary glands^22^.

### Peptide screening using a phage display library

Isolated 10^7^ primary hepatocytes were incubated with 2 × 10^11^ library phages^15^ for 30 min in a 1.5 ml tube with intermittent agitation. Hepatocytes were then transferred to a 15 ml tube and washed six times with 3 ml PBS. After the final wash, hepatocytes were resuspended in 3 ml of host *E. coli* (~10^8^ cells) culture in LB broth and incubated for 10 min at 37 °C to allow bound phages to infect host *E. coli*. Phages were amplified by culturing the infected bacteria overnight in 100 ml LB broth with tetracycline^15^. Amplified phages were precipitated by adding to the culture supernatant, the same volume of 16% PEG8000 (Fisher Scientific) in PBS. After three additional rounds of selection using fresh primary mouse hepatocytes, the selected bound phages were recovered, amplified in *E. coli*, and plated on a tetracycline LB agar plate. The peptide-coding DNA from 48 randomly picked phage colonies from two independent experiments was PCR amplified. Of these, 39 were successfully sequenced^15^.

### Recombinant protein expression

*P. berghei* sporozoite candidate proteins (Fig. 5a), pET-HP1 peptide (Fig. 7), and ~19 kDa empty pET tag protein (thioredoxin-tag, N- and C-terminal His-tags, and S-tag) were expressed using the pET32b expression system (Novagen) and BL21(DE3) *E. coli*. All DNA inserts for recombinant protein expression were synthesized by BioBasic Inc (www.biobasic.com). Protein expression was induced by the addition of 0.4 mM IPTG for 4 h. For purification of recombinant proteins, the induced cell pellet was lysed in 1% Triton X100. Cell lysates were incubated with S-tag agarose (Millipore) for the purification of recombinant proteins. Bound protein was eluted with low pH buffer and elution buffer was exchanged with PBS by dialysis.

### Immunofluorescence assays

Mammalian cells or sporozoites were fixed in 4% paraformaldehyde in PBS for 30 min and blocked with 4% BSA in PBS for 1 h. Mouse frozen liver sections were fixed in methanol and blocked with 4% BSA in PBS. Sporozoite-infected mammalian cells were permeabilized in 0.2% Triton X100 in PBS for 15 min after fixation. Immunofluorescence assays for sporozoite surface molecules used 0.5% antisera in PBS. Peptide or recombinant protein-binding assays used 200 μg/ml of peptide or recombinant protein. We used commercially available anti-S-tag (Novagen), anti-His tag (Genescript), anti-CPS1(Proteintech), anti-vWA8 (Invitrogen), and Alexa488-conjugated secondary (Invitrogen) antibodies under the manufacturer’s directions.

### In vitro sporozoite inhibition assays

For in vitro sporozoite inhibition assays, sporozoites were incubated with host cells (one sporozoite per two host cells) for 3 h allowing initial binding and invasion, and sporozoite attachment and invasion was measured. *P*. *berghei* experiments used freshly isolated 2 × 10^5^ primary mouse hepatocytes in 1.5 ml tube and *P. falciparum* experiments used two human hepatoma cell lines—HepG2 and Huh7—in eight-well chamber slide (Labtek-II), 10^5^ host cells per chamber were added for monolayer culture, to confirm that the results are not cell-specific. For inhibition assays, cells were incubated with 10^10^ phages/ml, 400 μg/ml peptide, 0.1–0.2 mg/ml purified antibody or 20–50% antisera in culture medium. Sporozoite–host cell interaction was determined by counting bound plus invaded sporozoites after triple washes with PBS in 20 microscopic fields under ×400 magnification with triplicated tubes, ~5 μl of cell suspension containing ~10^3^ mouse hepatocytes (Figs. 1, 2a, and 6b), or chambers, human hepatoma cell monolayer (Figs. 2b,  3b right panel,  6d, Supplementary Fig. 7c), per treatment which covers ~40% assayed cells. Bound and invaded sporozoites were identified with an anti-CSP antibody and Alexa488-conjugated secondary antibody after permeabilization. Relative sporozoite interaction was determined by normalizing sporozoite counts comparing to the mean of control group.

### Immunization and antibodies

For immunization and challenge assays, 21–25 g female Swiss Webster mice were injected with 50 μg per mouse of each KLH-conjugated peptide or with KLH alone using 50% AddaVax adjuvant (InvivoGen). Priming subcutaneous injection was followed by triple boosts at two-week intervals. At 10 days after the last boost, immunized mice were challenged with *P. berghei* sporozoites via biting by two infected *An. stephensi* mosquitoes. For mouse challenge assays, highly infected female mosquitoes were selected by examination of salivary gland fluorescence, 18 days after mosquito feeding on mice infected with tdTomato-expressing parasites^24^. Mouse parasite infection was determined by thin blood smears followed by Giemsa staining from days 4 to 12 post-challenge. Polyclonal anti-PbPLS or anti-pET tag antisera were generated by immunization of mice with 50 μg of recombinant protein per mouse with 50% AddaVax adjuvant (InvivoGen).

### Western and far-Western blotting assays

Parasites, host cells, and recombinant proteins were harvested, pelleted, resuspended with 1× SDS gel loading buffer, and boiled for 10 min. Protein lysates were fractionated by 10% SDS–PAGE and then stained with Coomassie dye or transferred onto a PVDF membrane. Membranes were incubated for 1 h in blocking buffer (0.1% Tween 20 and 4% milk powder in PBS), followed by the addition of a primary antibody. Antibody binding was visualized with alkaline phosphatase-conjugated secondary antibody (Promega). For the far-Western blotting assay in Fig. 7a, mouse hepatocytes were used for sub-cellular fractionation as previously described^8^. A total of 1–2 × 10^7^ primary mouse hepatocytes were harvested with buffer 1 (0.01% digitonin, 10 mM PIPES, pH 6.8, 300 mM sucrose, 100 mM NaCl, 3 mM MgCl_2_, and 5 mM EDTA) and rotated for 10 min at 4 °C. After 1-min centrifugation (16,900 × *g*), the supernatant (cytosolic fraction) was collected, and the cell pellet was washed with buffer 1. The pellet was then resuspended with buffer 2 (0.5% Triton X-100, 10 mM PIPES, pH 7.4, 300 mM sucrose, 100 mM NaCl, 3 mM MgCl_2_, and 3 mM EDTA) and rotated for 20 min at 4 °C. After 1-min centrifugation (16,900 × *g*), the supernatant (membrane fraction) was collected, and the cell pellet was washed with buffer 2. The pellet was then resuspended in 6.5 M urea (insoluble fraction). Each fraction was fractionated by 10% SDS–PAGE and transferred onto a PVDF membrane. The membrane was incubated in 0.1 mg/ml of recombinant pET tag or pET-HP1 peptide. Protein binding was detected with an anti-S-tag antibody.

### ELISA

Recombinant protein or biotinylated peptide was attached to a Ni-coated or a streptavidin-coated ELISA plate (Thermo Fisher), respectively. After washing and blocking with 1% BSA in PBS, the coated wells were further incubated with antibody or hepatocyte lysate. An anti-S tag antibody determined the amount of recombinant protein bound to the Ni-coated well for normalization. Anti-HP1 antibody binding was quantified by alkaline phosphatase-conjugated secondary antibody (Supplementary Figs. 4 and 5) and binding hepatocyte proteins CPS1 or VWA8 to PLS was quantified by using a non-tagged primary antibody and alkaline phosphatase-conjugated secondary antibodies (Fig. 8c).

### Pull-down assays

For pull-down assays (Figs. 7b and 8b), recombinant pET-HP1, pET-PLS, or pET tag protein was bound to S-tag agarose beads (Millipore) and incubated overnight with hepatocyte lysate in 1% Triton X100. After washing, bound proteins were eluted by boiling S-tag agarose beads in 1× SDS gel loading buffer. Western blotting assays with an anti-CPS1 or anti-VWA8 antibody visualized bound protein to the beads.

### Statistical analyses

For ELISA assays we used the one-way ANOVA Tukey honest significant differences (Tukey HSD) test for two tail *P-*value using SPSS (12.0) software. For Kaplan–Meier test we used GraphPad Prism (9.2.0) for a two-tail *P-*value. For infection and inhibition assays we used Mann–Whitney *U* test for the two-tail *P-*value. The number of independent repeats is specified in the legends.

### Reporting summary

Further information on research design is available in the Nature Research Reporting Summary linked to this article.

## Supplementary information

Supplementary Information

Reporting Summary

## Data Availability

All experimental data were listed accordingly. Source data are provided with this paper as Source Data files. Detailed protein information in Tables 1 and 2 can be found in https://plasmodb.org/plasmo/app/record/gene/PBANKA_0510200, https://plasmodb.org/plasmo/app/record/gene/PBANKA_1422900, https://plasmodb.org/plasmo/app/record/gene/PBANKA_1034500, https://plasmodb.org/plasmo/app/record/gene/PBANKA_1240600, https://plasmodb.org/plasmo/app/record/gene/PBANKA_1026200, https://plasmodb.org/plasmo/app/record/gene/PBANKA_0506900, https://plasmodb.org/plasmo/app/record/gene/PBANKA_0403200, and https://www.uniprot.org/uniprot/Q8C196, https://www.uniprot.org/uniprot/Q8CC88, https://www.uniprot.org/uniprot/Q9DBM2, https://www.uniprot.org/uniprot/Q6PB66, https://www.uniprot.org/uniprot/F8WHL2. Source data are provided with this paper.

## Source data

Source Data
