# Supplementary material for: Plasmodium sporozoite phospholipid scramblase interacts with mammalian carbamoyl-phosphate synthetase 1 to infect hepatocytes
Source: Nat Commun. 2021 Nov 19;12:6773. doi: 10.1038/s41467-021-27109-7 (PMC8604956; doi:10.1038/s41467-021-27109-7)

SUPPLEMENTARY INFORMATION

Plasmodium sporozoite phospholipid scramblase interacts with mammalian carbamoyl-phosphate synthetase 1 to infect hepatocytes

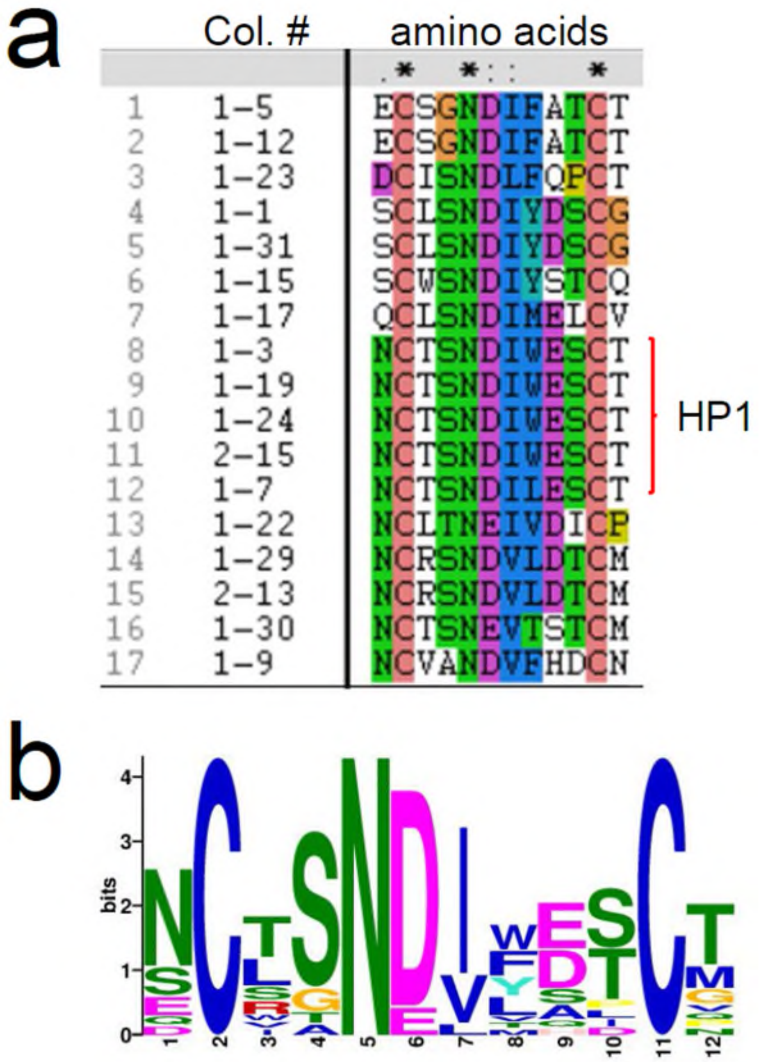

Supplementary Fig. 1| Selection of hepatocyte binding peptide. a, A total of 39 successfully sequenced recombinant phages from two independent screens (four rounds of selection each) were sorted by displayed peptide sequence using Clustal X (1.81) multiple sequence alignment (http://www.clustal.org/clustal2/). Of the 39 peptides, 17 (43.6%) had the most related sequences. Out of these, five peptides had identical sequence that was termed HP1. **b,** MEME analysis (https://meme-

suite.org/meme/tools/meme) shows the most common amino acids at each position[1]. Col.#: experiment number-colony number; *, fully conserved residue; :, strongly conserved residue; ., weakly conserved residue.

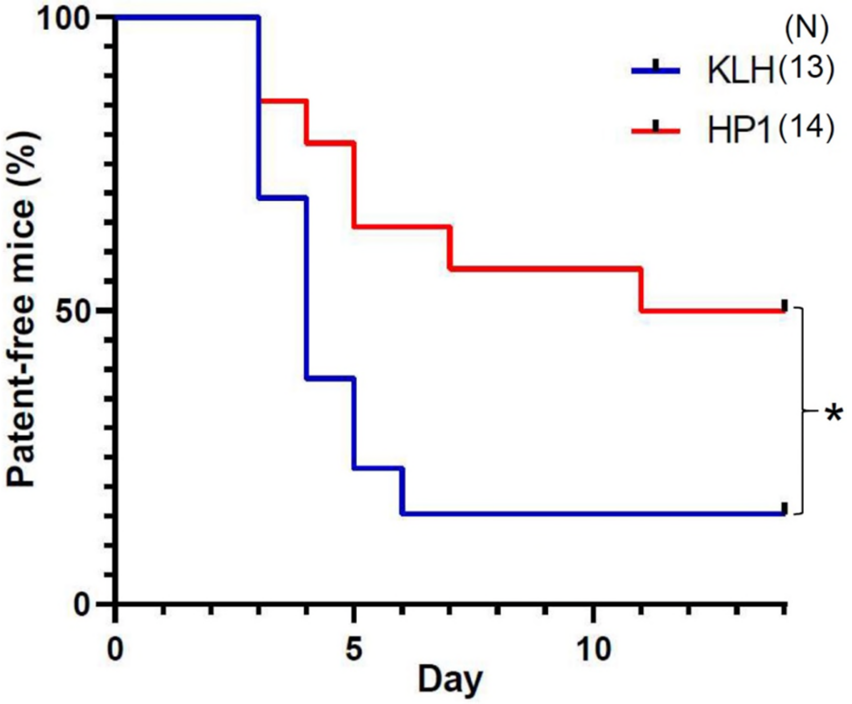

Supplementary Fig. 2| Kaplan-Meier analysis shows HP1 immunization inhibits sporozoite liver infection (related to Fig. 3b). Mice immunized with KLH-conjugated HP1 peptide or unconjugated KLH were challenged by the bite of two infected mosquitos. Thin blood smear and Giemsa stain determined parasite infection status. Prepatent day was compared by Kaplan-Meier (log-rank test) analysis (*, P=0.03). The comparison shows that HP1 immunization inhibited sporozoite liver infection (with 41 % efficacy) and delayed the prepatent period by one day (5 days, median) compared to the KLH-immunized control group (4 days, median). Data pooled from three independent experiments. N, number of total mice. Two tail P-value was calculated with Log-rank (Mantel-Cox) test. *, $P<0.05$. Source data are provided as a Source Data file.

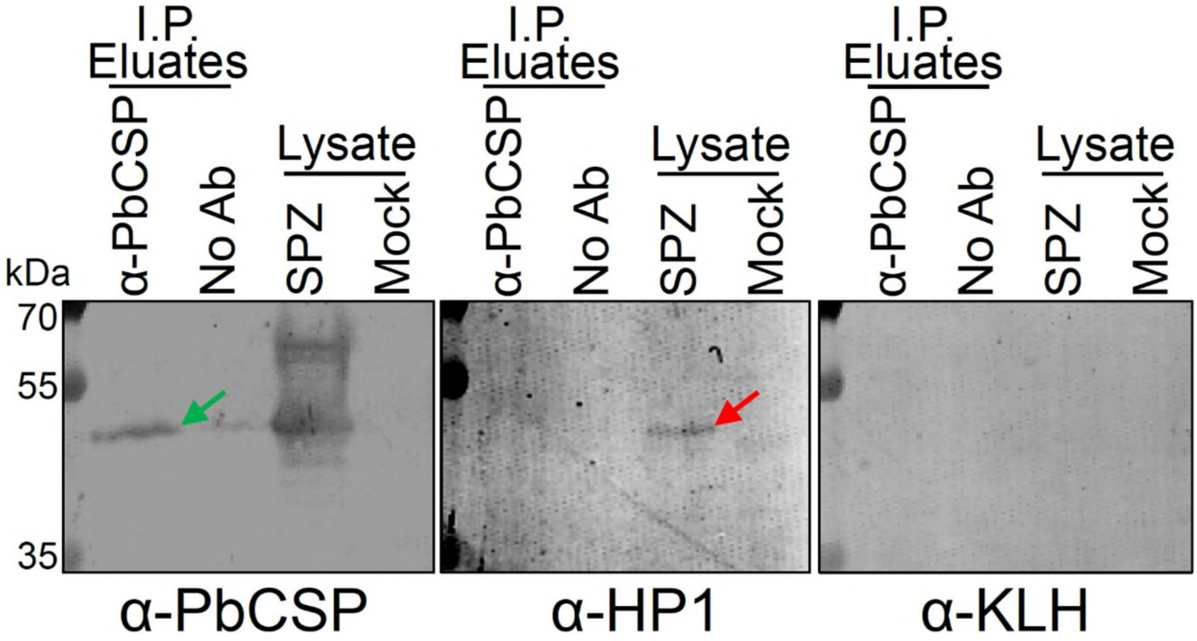

Supplementary Fig. 3| The anti-HP1 antibody does not recognize the sporozoite CSP protein. *P. berghei* sporozoite lysates (SPZ) were immunoprecipitated with an anti-PbCSP monoclonal antibody or in the absence of antibody as control. Each immunoprecipitation eluate (2 lanes) and lysates from sporozoites (SPZ) & mock preparations from non-infected salivary glands (2 lanes) were fractionated by SDS-PAGE. Western blotting with the antibody denoted below each panel showed that PbCSP was precipitated (green arrow), and that the anti-HP1 antibody did not recognize the immunoprecipitated protein, but recognized a ~50 kDa sporozoite protein present in the sporozoite lysate (red arrow). Each image represents two independent repeats. Source data are provided as a Source Data file.

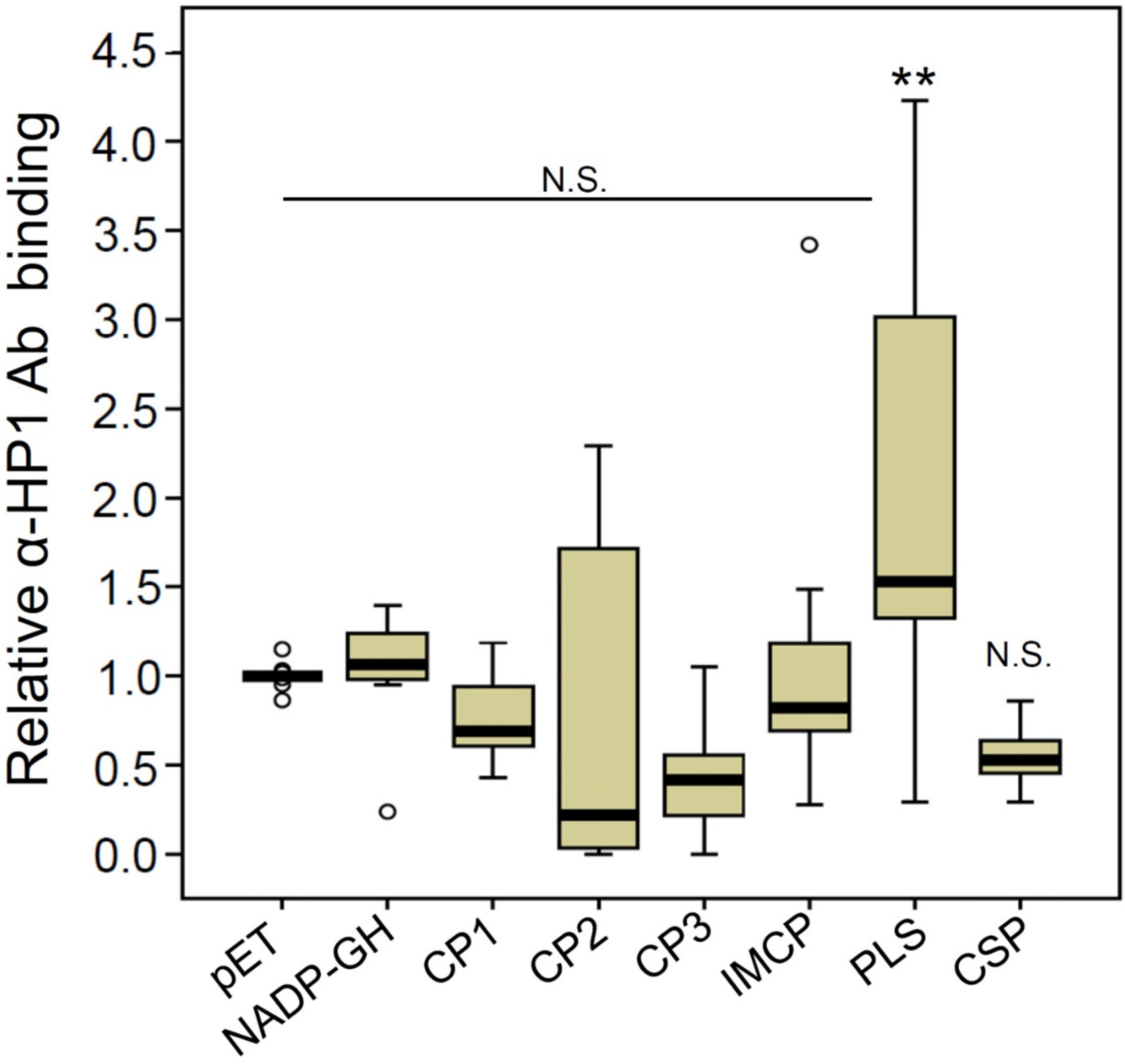

Supplementary Fig. 4| ELISA assays show that the anti-HP1 antibody recognizes recombinant PLS. Ni-coated ELISA plate was incubated overnight with the seven recombinant proteins listed in Table 1 and with the pET tag protein as a control. After blocking, 0.5 % anti-HP1 serum in PBS or anti-S-tag antibody (to estimate amount of recombinant protein bound) were added to each well. Antibody binding was determined with alkaline phosphatase-conjugated secondary antibody and colorimetric substrate. Anti-HP1 antibody binding intensity was normalized to anti-S-tag antibody binding (**, P=0.007; N.S., not significant). Data pooled from four independent repeats. All box plots

show quartiles, medians, and maximum within the bounds of 1.5 times interquartile range. Dots represent outliers beyond the upper and lower bounds. Two tail *P*-value was calculated with one-way ANOVA TukeyHSD test. Source data are provided as a Source Data file.

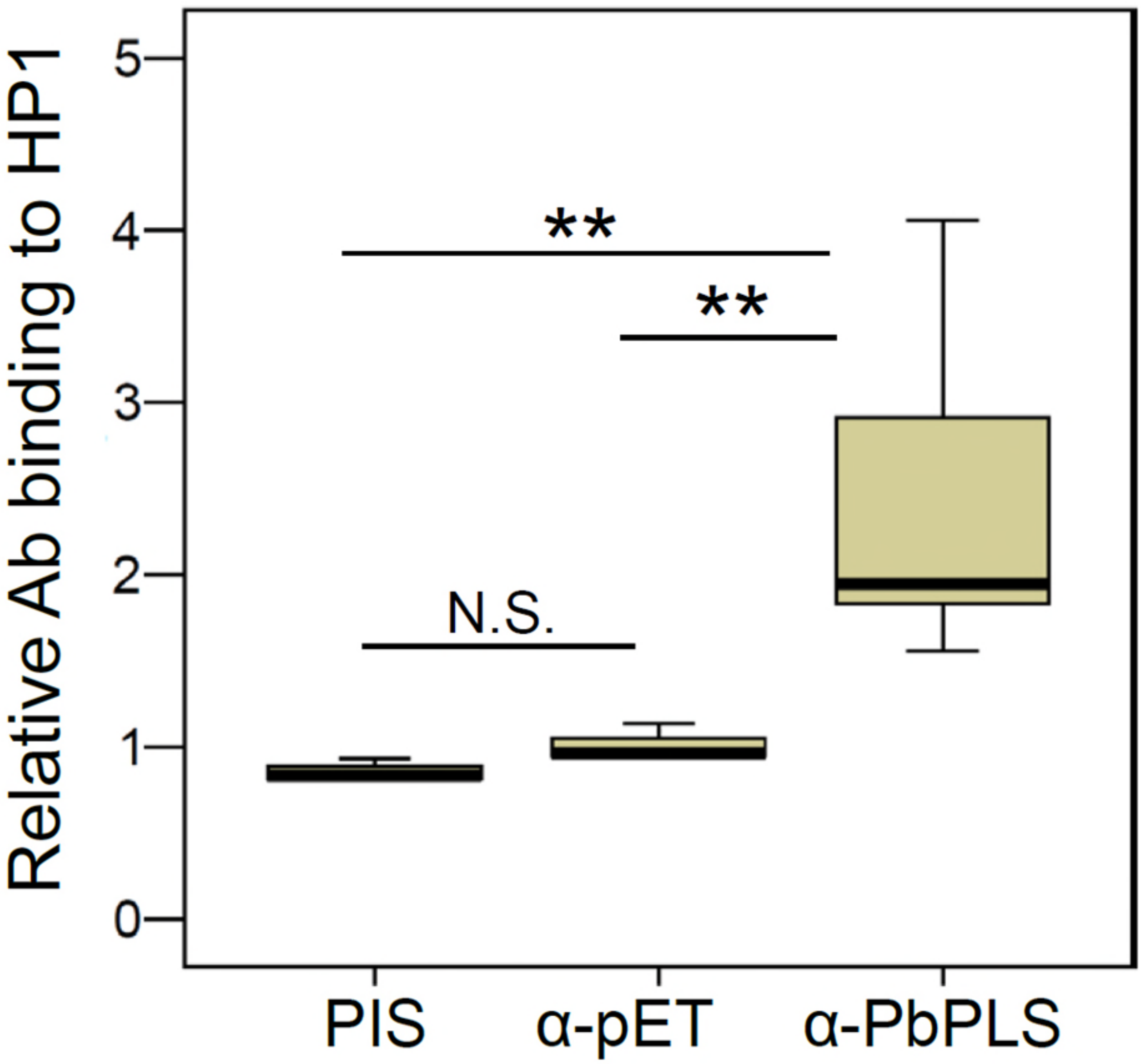

Supplementary Fig. 5| ELISA assays show that the anti-PLS antibody cross reacts with the HP1 peptide. Streptavidin-coated ELISA plates were incubated with biotinylated HP1 peptide and after blocking, 0.5 % anti-PLS, or anti-pET and preimmune sera (PIS) as controls, were added to separate wells. Antibody binding was determined with alkaline phosphatase-conjugated secondary antibody and colorimetric substrate. Mean anti-PLS antibody binding intensity relative to anti-pET antibody binding was determined (**, upper P= 0.004. lower P= 0.008; N.S., not significant). Data pooled from two independent experiments. Total of four PIS sera, four anti-pET sera, and ten anti-PLS sera samples were independently tested. All box plots show quartiles, medians, and maximum within the

bounds of 1.5 times interquartile range. Two tail *P*-value was calculated with one-way ANOVA TukeyHSD test.. Source data are provided as a Source Data file.

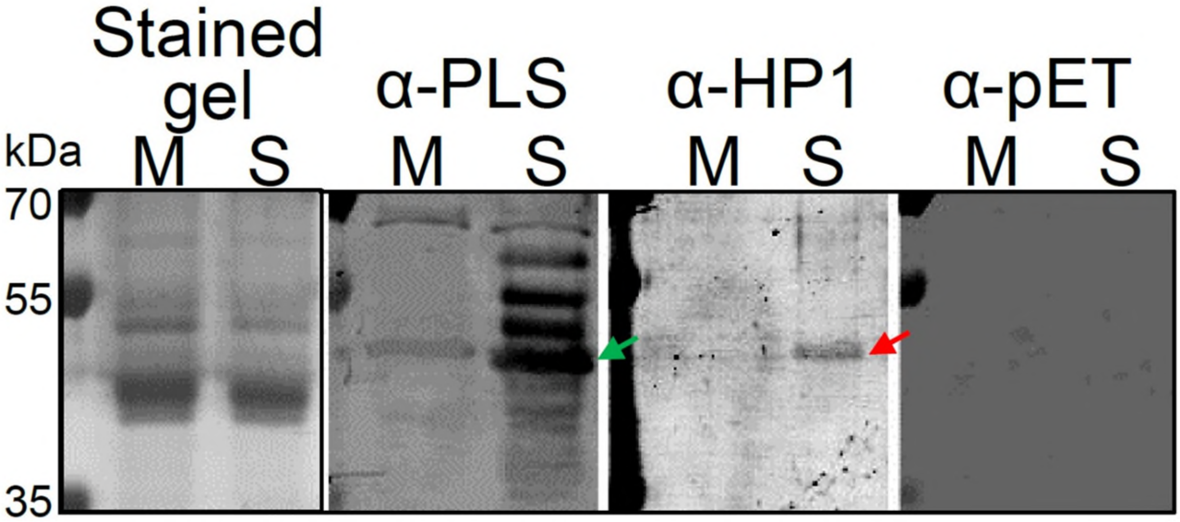

Supplementary Fig. 6| An anti-PbPLS serum and an anti-HP1 serum recognize proteins with the same mobility. Lysates of salivary gland sporozoites (S) or mock preparations (M) were fractionated by SDS-PAGE and transferred onto PVDF membranes. Western blotting shows a ~50 kDa protein band recognized both, by the anti-PLS antibody (green arrow) and the anti-HP1 antibody (red arrow), whereas the anti-pET antiserum does not recognize the band. S: lysate of sporozoites from infected salivary glands; M: lysate from non-infected salivary glands (mock preparations). Each image represents two independent repeats. Source data are provided as a Source Data file.

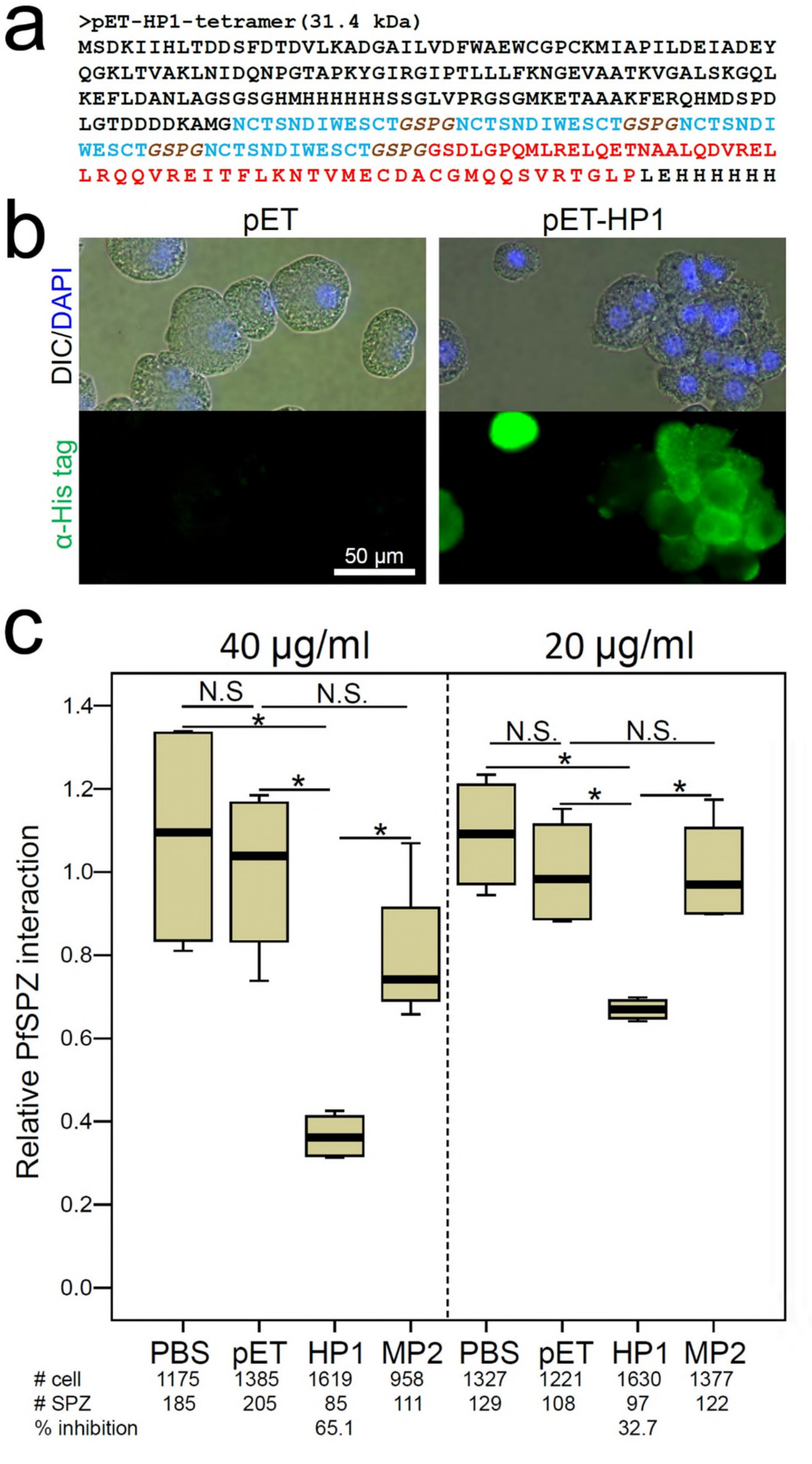
a
>pET-HP1-tetramer(31.4 kDa)
MSDKIIHLTDDSFDTDVLKADGAILVDFWAEWCGPCKMIAPILDEIADEY
QGKLTVAKLNIDQNPGTAPKYGIRGIPTLLLFKNGEVAATKVGALSKGQL
KEFLDANLAGSGSGHMHHHHHHSSGLVPRGSGMKETAAAKFERQHMDSPD
LGTDDDDKAMGNCTSNDIWESCTGSPGNCTSNDIWESCTGSPGNCTSNDI
WESCTGSPGNCTSNDIWESCTGSPGGSDLGPQMLRELQETNAALQDVREL
LRQQVREITFLKNTVMECDACGMQQSVRTGLPLEHHHHHH
b
pET
pET-HP1
DIC/DAPI
α-His tag
50 μm
c
40 μg/ml
20 μg/ml
Relative PfSPZ interaction
N.S
N.S.
*
N.S.
N.S.
1.4
1.2
1.0
0.8
0.6
0.4
0.2
0.0
PBS
pET
HP1
MP2
PBS
pET
HP1
MP2
cell 1175 1385 1619 958 1327 1221 1630 1377
SPZ 185 205 85 111 129 108 97 122
% inhibition 65.1 32.7

Supplementary Fig. 7| A recombinant tetrameric HP1 peptide, part of a five-stranded alpha-helical bundle, binds to hepatocytes and inhibits sporozoite-hepatocyte interaction. a, Amino acid sequence of the recombinant tetrameric HP1 peptide (blue font) separated by four-amino-acid linkers (brown italic font), fused to a five-stranded alpha-helical bundle (red font)[2]. Black font shows amino acid sequence of pET32b expression tag including a thioredoxin, a S-tag, two 6 x His tags, Recombinant pET tag protein or pET tagged recombinant HP1 peptide were incubated with mouse primary hepatocytes. Protein binding was visualized with anti-His tag antibody and Alexa488-conjugated secondary antibody. DIC: Differential Interference Contrast microscopy; DAPI: nuclear stain (blue). Each image represents two independent repeats. **c**, Recombinant pET tag protein, tetrameric HP1 peptide, or tetrameric MP2 peptide[3] (unrelated control that binds to mosquito midgut epithelium) at 40 or 20 μg/ml final concentration, were added to a HepG2 cell culture together with *P. falciparum* sporozoites. Relative sporozoite-hepatocyte interaction was determined by counting the number of sporozoite-bound and -invaded cells/total hepatocyte number. An anti-CSP antibody and Alexa488-conjugated secondary antibody were used after permeabilization to detect sporozoites. Recombinant tetrameric HP1 peptide inhibits sporozoite-hepatocyte interaction in a dose-dependent manner. % inhibition was calculated using medians. Relative sporozoite interaction was determined by normalizing sporozoite counts comparing to the mean of the PBS treated control group (***,** all P = 0.02; N.S., not significant). Data pooled of two independent experiments. All box plots show quartiles, medians, and maximum within the bounds of 1.5 times interquartile range. Two tail P-value by Mann-Whitney U test. # cell: total hepatocyte number counted; # spz: total sporozoite number counted. Source data are provided as a Source Data file.

SUPPLEMENTARY REFERENCES.

1 Bailey, T. L. & Elkan, C. Fitting a mixture model by expectation maximization to discover motifs in biopolymers. *Proc Int Conf Intell Syst Mol Biol* **2**, 28-36 (1994).

2 Efimov, V. P., Lustig, A. & Engel, J. The thrombospondin-like chains of cartilage oligomeric matrix protein are assembled by a five-stranded alpha-helical bundle between residues 20 and 83. *FEBS Lett* **341**, 54-58, doi:10.1016/0014-5793(94)80239-4 (1994).

3 Vega-Rodriguez, J. *et al.* Multiple pathways for Plasmodium ookinete invasion of the mosquito midgut. *Proc Natl Acad Sci U S A* **111**, E492-500, doi:10.1073/pnas.1315517111 (2014).

Source Data Files

Supplementary Fig 3

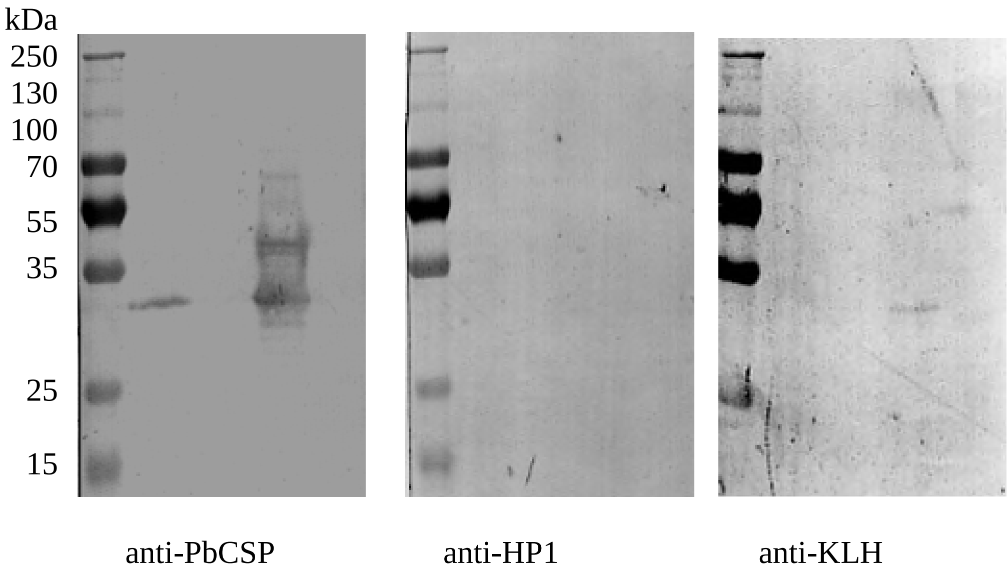

Supplementary Fig 6

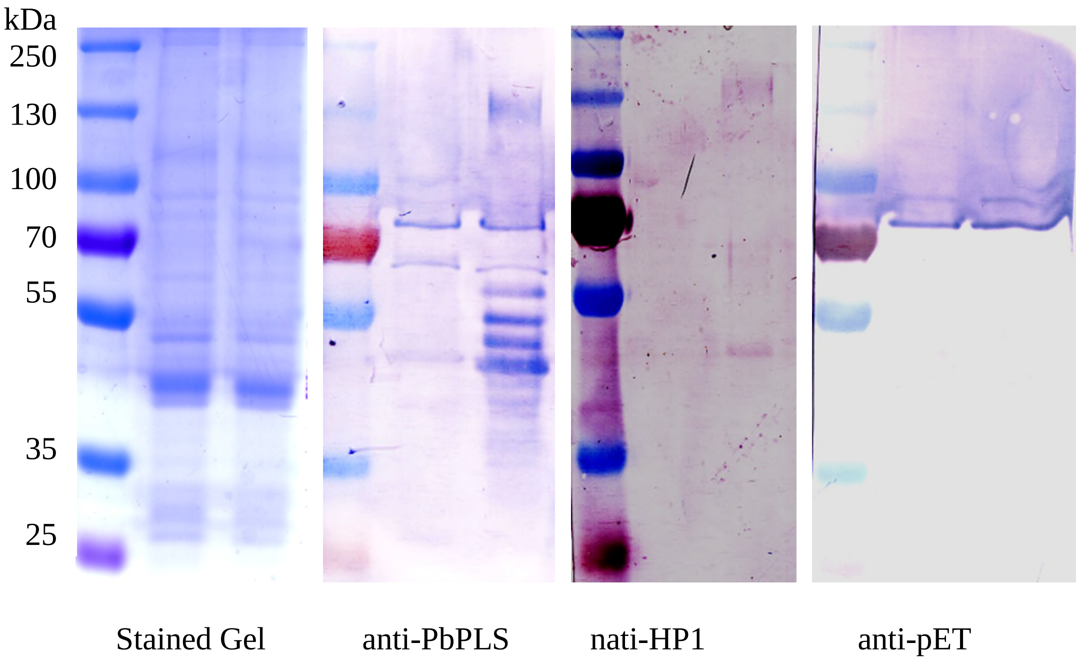

Supplement: Supplementary file 1 — Supplementary Information [file 41467_2021_27109_MOESM1_ESM.pdf]
