# Supplementary material for: Plasmodium sporozoite phospholipid scramblase interacts with mammalian carbamoyl-phosphate synthetase 1 to infect hepatocytes
Source: Nat Commun. 2021 Nov 19;12:6773. doi: 10.1038/s41467-021-27109-7 (PMC8604956; doi:10.1038/s41467-021-27109-7)
Supplement: Supplementary file 2 — Reporting Summary [file 41467_2021_27109_MOESM2_ESM.pdf]

nature portfolio

Corresponding author(s): Marcelo Jacobs-Lorena, Sung-Jae Cha

Last updated by author(s): Oct 26, 2021

Reporting Summary

Nature Portfolio wishes to improve the reproducibility of the work that we publish. This form provides structure for consistency and transparency in reporting. For further information on Nature Portfolio policies, see our Editorial Policies and the Editorial Policy Checklist.

Statistics

For all statistical analyses, confirm that the following items are present in the figure legend, table legend, main text, or Methods section.

n/a	Confirmed	
☐	☒	The exact sample size (n) for each experimental group/condition, given as a discrete number and unit of measurement
☐	☒	A statement on whether measurements were taken from distinct samples or whether the same sample was measured repeatedly
☐	☒	The statistical test(s) used AND whether they are one- or two-sided<br>*Only common tests should be described solely by name; describe more complex techniques in the Methods section.*
☒	☐	A description of all covariates tested
☒	☐	A description of any assumptions or corrections, such as tests of normality and adjustment for multiple comparisons
☐	☒	A full description of the statistical parameters including central tendency (e.g. means) or other basic estimates (e.g. regression coefficient) AND variation (e.g. standard deviation) or associated estimates of uncertainty (e.g. confidence intervals)
☐	☒	For null hypothesis testing, the test statistic (e.g. F, t, r) with confidence intervals, effect sizes, degrees of freedom and P value noted<br>*Give P values as exact values whenever suitable.*
☒	☐	For Bayesian analysis, information on the choice of priors and Markov chain Monte Carlo settings
☒	☐	For hierarchical and complex designs, identification of the appropriate level for tests and full reporting of outcomes
☒	☐	Estimates of effect sizes (e.g. Cohen's d, Pearson's r), indicating how they were calculated

Our web collection on statistics for biologists contains articles on many of the points above.

Software and code

Policy information about availability of computer code

Data collection	no software was used
Data analysis	Statistical analyses were performed with the SPSS (12.0) software. For Kaplan-Meier test we used GraphPad Prism (9.2.0) soft ware. Multiple sequence alignment was performed with the Clustal X (1.81) software.

For manuscripts utilizing custom algorithms or software that are central to the research but not yet described in published literature, software must be made available to editors and reviewers. We strongly encourage code deposition in a community repository (e.g. GitHub). See the Nature Portfolio guidelines for submitting code & software for further information.

Data

Policy information about availability of data

All manuscripts must include a data availability statement. This statement should provide the following information, where applicable:

- Accession codes, unique identifiers, or web links for publicly available datasets
- A description of any restrictions on data availability
- For clinical datasets or third party data, please ensure that the statement adheres to our policy

All experimental data were listed accordingly. Source data are provided with this paper as Source Data files. Detailed protein information in Table 1 and 2 can be found in https://plasmodb.org/plasmo/app and https://www.uniprot.org/ using PlasmoDB ID and Uniprot ID, respectively.

Field-specific reporting

Please select the one below that is the best fit for your research. If you are not sure, read the appropriate sections before making your selection.

- [x] Life sciences
- [ ] Behavioural & social sciences
- [ ] Ecological, evolutionary & environmental sciences

For a reference copy of the document with all sections, see nature.com/documents/nr-reporting-summary-flat.pdf

Life sciences study design

All studies must disclose on these points even when the disclosure is negative.

Sample size	Each experimental sample size was determined by multiple biological repeats that allow analysis of sufficient statistical significance. Sample size is reported for each experiment.
Data exclusions	No data were excluded.
Replication	We performed all experiments with at least two and more independent experiments. Data from each experimental replication showed similar trend and were pooled for statistical analysis.
Randomization	Reported experiments did not require randomization because of using only homogeneous experimental animals or animal cells.
Blinding	Blinding is not relevant to the experiments reported in this study because of using only homogeneous experimental animals or animal cells.

Reporting for specific materials, systems and methods

We require information from authors about some types of materials, experimental systems and methods used in many studies. Here, indicate whether each material, system or method listed is relevant to your study. If you are not sure if a list item applies to your research, read the appropriate section before selecting a response.

Materials & experimental systems

n/a	Involved in the study	
[ ]	[x]	Antibodies
[ ]	[x]	Eukaryotic cell lines
[x]	[ ]	Palaeontology and archaeology
[ ]	[x]	Animals and other organisms
[x]	[ ]	Human research participants
[x]	[ ]	Clinical data
[x]	[ ]	Dual use research of concern

Methods

n/a	Involved in the study	
[x]	[ ]	ChIP-seq
[x]	[ ]	Flow cytometry
[x]	[ ]	MRI-based neuroimaging

Antibodies

Antibodies used	1. Commercially available antibodies: anti-S-tag (Novagen, 71549), anti-His tag (Genescript, A00186-100), anti-CPS1(Proteintech, 18703-1-AP), anti-vWA8 (Invitrogen, PA5-58648), and Alexa488-conjugated secondary (Invitrogen, A32723 , A32731) antibodies, alkaline phosphatase–conjugated secondary antibody (Promega, S3721, S3731) under manufacturer's directions<br>2. Monoclonal antibodies against Plasmodium sporozoite circumsporozoite protein: anti-PbCSP (3d11) and anti-PfCSP (2a10) at 0.5 microgram per ml in PBS<br>3. Anti-sera collected from immunized mice were diluted to 5 % in PBS for immunofluorescent and Western blotting assays, or 0.1-0.2 mg/ml purified antibody or 20-50 % antisera in culture medium for inhibition assay: anti-HP1 phage anti-sera, anti-WT phage anti-sera, anti-HP1 peptide antibody, anti-PbPLS antibody, anti-pET antibody
Validation	1. Anti-CPS1 antibody reacts with mouse CPS1 (https://www.ptglab.com/products/CPS1-Antibody-18703-1-AP.htm)<br>2. Anti-vWA8 antibody was predicted to interact with mouse vWA8 based on the 89% antigen sequence identity (https://www.thermofisher.com/antibody/product/VWA8-Antibody-Polyclonal/PA5-58648)<br>3. Anti-PfCSP 2a10 monoclonal antibody (Product Information Sheet for MRA-183A at https://www.beiresources.org/) and anti-PbCSP 3d11 monoclonal antibody (Yoshida et al. 1980, http://dx.doi.org/10.1126/science.6985745) bind to sporozoite surface CSP protein.<br>4. Validation of anti-sera collected from immunized mice in this study can be found in Fig 3, 4, 5, 6 and Supplementary Fig 3, 4, 5, 6.

Eukaryotic cell lines

Policy information about cell lines

Cell line source(s)	Human hepatoma cell lines: Huh7 (ThermoFisher), HepG2 (ATCC)
Authentication	This study has no cell line authentication related data.
Mycoplasma contamination	The cell lines were not tested for mycoplasma contamination.
Commonly misidentified lines (See ICLAC register)	No commonly misidentified cell lines were used in the study.

Animals and other organisms

Policy information about studies involving animals; ARRIVE guidelines recommended for reporting animal research

Laboratory animals	Four- to six-week old outbred female Swiss Webster or male and female C57BL/6J mice were used for rodent malaria parasite Plasmodium berghei infection. Parasites were maintained in the same mouse strains or Anopheles stephensi mosquito. Mouse rooms are maintained at 30-70% relative humidity and a temperature of 18-26ºC (64-79ºF) providing 14 hours light/10 hours dark.
Wild animals	The study did not involve wild animals
Field-collected samples	The study did not involved field-collected samples
Ethics oversight	Johns Hopkins University Animal Care and Use Committee (ACUC) approved our experimental mouse protocol.

Note that full information on the approval of the study protocol must also be provided in the manuscript.
